# Quantifying Missing Heritability at Known GWAS Loci

**DOI:** 10.1371/journal.pgen.1003993

**Published:** 2013-12-26

**Authors:** Alexander Gusev, Gaurav Bhatia, Noah Zaitlen, Bjarni J. Vilhjalmsson, Dorothée Diogo, Eli A. Stahl, Peter K. Gregersen, Jane Worthington, Lars Klareskog, Soumya Raychaudhuri, Robert M. Plenge, Bogdan Pasaniuc, Alkes L. Price

**Affiliations:** 1 Department of Epidemiology, Harvard School of Public Health, Boston, Massachusetts, United States of America; 2 Department of Biostatistics, Harvard School of Public Health, Boston, Massachusetts, United States of America; 3 Medical and Population Genetics Program, Broad Institute, Cambridge, Massachusetts, United States of America; 4 Harvard-Massachusetts Institute of Technology (MIT) Division of Health, Science and Technology, Cambridge, Massachusetts, United States of America; 5 Department of Medicine Lung Biology Center, University of California San Francisco, San Francisco, California, United States of America; 6 Division of Rheumatology, Immunology, and Allergy, Brigham and Women's Hospital, Harvard Medical School, Boston, Massachusetts, United States of America; 7 Division of Genetics, Brigham and Women's Hospital, Harvard Medical School, Boston, Massachusetts, United States of America; 8 The Feinstein Institute for Medical Research, North Shore-Long Island Jewish Health System, Manhasset, New York, United States of America; 9 Arthritis Research UK Epidemiology Unit, University of Manchester, Manchester Academic Health Sciences Centre, Manchester, United Kingdom; 10 Rheumatology Unit, Department of Medicine, Karolinska Institutet and Karolinska University Hospital Solna, Stockholm, Sweden; 11 Department of Pathology and Laboratory Medicine, Geffen School of Medicine at UCLA, Los Angeles, California, United States of America; The University of Queensland, Australia

## Abstract

Recent work has shown that much of the missing heritability of complex traits can be resolved by estimates of heritability explained by all genotyped SNPs. However, it is currently unknown how much heritability is missing due to poor tagging or additional causal variants at known GWAS loci. Here, we use variance components to quantify the heritability explained by all SNPs at known GWAS loci in nine diseases from WTCCC1 and WTCCC2. After accounting for expectation, we observed all SNPs at known GWAS loci to explain 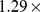 more heritability than GWAS-associated SNPs on average (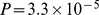). For some diseases, this increase was individually significant: 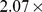 for Multiple Sclerosis (MS) (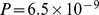) and 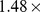 for Crohn's Disease (CD) (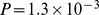); all analyses of autoimmune diseases excluded the well-studied MHC region. Additionally, we found that GWAS loci from other related traits also explained significant heritability. The union of all autoimmune disease loci explained 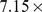 more MS heritability than known MS SNPs (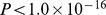) and 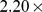 more CD heritability than known CD SNPs (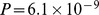), with an analogous increase for all autoimmune diseases analyzed. We also observed significant increases in an analysis of 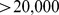 Rheumatoid Arthritis (RA) samples typed on ImmunoChip, with 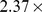 more heritability from all SNPs at GWAS loci (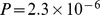) and 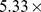 more heritability from all autoimmune disease loci (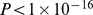) compared to known RA SNPs (including those identified in this cohort). Our methods adjust for LD between SNPs, which can bias standard estimates of heritability from SNPs even if all causal variants are typed. By comparing adjusted estimates, we hypothesize that the genome-wide distribution of causal variants is enriched for low-frequency alleles, but that causal variants at known GWAS loci are skewed towards common alleles. These findings have important ramifications for fine-mapping study design and our understanding of complex disease architecture.

## Author Summary

Heritable diseases have an unknown underlying “genetic architecture” that defines the distribution of effect-sizes for disease-causing mutations. Understanding this genetic architecture is an important first step in designing disease-mapping studies, and many theories have been developed on the nature of this distribution. Here, we evaluate the hypothesis that additional heritable variation lies at previously known associated loci but is not fully explained by the single most associated marker. We develop methods based on variance-components analysis to quantify this type of “local” heritability, demonstrating that standard strategies can be falsely inflated or deflated due to correlation between neighboring markers and propose a robust adjustment. In analysis of nine common diseases we find a significant average increase of local heritability, consistent with multiple common causal variants at an average locus. Intriguingly, for autoimmune diseases we also observe significant local heritability in loci not associated with the specific disease but with other autoimmune diseases, implying a highly correlated underlying disease architecture. These findings have important implications to the design of future studies and our general understanding of common disease.

## Introduction

While association studies have been successful in finding a large number of significant variants for many complex traits, they have individually explained relatively little of the total heritability, motivating analyses that seek to identify this so-called “missing” heritability [1]–[3]. One hypothesis is that additional causal variation is present at the known GWAS loci but not fully quantified by individual GWAS markers [1], [2], [4]–[7]. This scenario may arise if the true causal variant is poorly tagged by any single GWAS marker [8] or if multiple independent causal variants exist at the locus [9]. In this case, the variance explained by the most-significant marker would only provide a lower bound on the local contribution, and some of the “missing” heritability would in fact be hidden at the previously discovered loci. If we consider “local” heritability to be the measure of aggregate variance from all causal variants at a locus, its quantification is an important step towards fully understanding the contributions made by association studies. Moreover, estimating components of local heritability indirectly from the vast amount of GWAS-level data already available would enrich our current understanding of complex disease architecture and provide insights into further study-design for post-GWAS fine-mapping studies. Here, we investigate methods for inferring components of local heritability at previously identified GWAS loci.

As study sample sizes continue to grow, researchers have focused on quantifying the amount of heritability explained by individually significant single-marker associations [4], [10]–[14]. In well-powered GWAS, one can also look for secondary variants that are conditionally independent of the leading SNP and estimate the joint contribution to phenotype. This conditional analysis has recently proven effective in GWAS for height [4], [15], [16] and multiple case-control traits [17], where a handful of loci were found to contain independent secondary associations. This strategy inherently focuses on a small number of independent markers and the outcome strongly depends on power to detect the primary association as well as any secondary variants. Such complexities make it difficult to compare this estimate across different studies and disease architectures. With additional resources, one can fine-map implicated loci using denser genotyping or sequencing platforms and look for more strongly significant markers. Recent studies involving re-sequencing around known GWAS-associated regions have identified additional variants explaining significant heritability in several complex traits [5], [18]–[20]. Looking beyond individual traits, a fine-mapping study of Celiac disease examined loci associated with other autoimmune diseases and nearly doubled the number of significant associations [21]. This approach can leverage the shared genetic architecture observed in some groups of related traits [22]–[24]. Still, such studies have not always yielded significant associations; a targeted re-sequencing analysis of Type 2 Diabetes did not yield any additional variants beyond what was known from GWAS [25] and recent work with dense genotyping did not uncover significant additional heritability at known loci for Type 2 Diabetes and Coronary Artery Disease [20]. Overall, these findings motivate methods that can infer components of additional local heritability using available GWAS data to guide fine-mapping analysis for identifying additional risk variants.

We propose to address this challenge by making use of all observed markers in a variance-component analysis, which optimizes a single measure of effect-size over a sample relatedness matrix. When sample relatedness is computed directly from the observed markers - referred to as the genetic relatedness matrix (GRM) - this variance-component can be used to infer the narrow-sense heritability explained by these markers. This measurement of narrow-sense heritability represents the aggregate effect of all causal variants observed or tagged in the data assuming additive, normally-distributed effect sizes. Recent work in variance-components analysis has shown that the contribution of all genotyped SNPs and any markers in LD with them, denoted 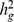, can be estimated directly from large-sample GWAS data in this way [26]–[29]. Similarly, our aim is to apply the variance-component model locally, by constructing the GRM from all typed SNPs at known GWAS regions and estimating the corresponding local 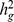. The excess of this quantity over the variance explained by known associations provides a lower bound on additional heritability at the locus. Uniquely, this method allows the analysis of loci that have no known association in the focal trait but have been associated with other related traits, quantifying sources of missing heritability implicated by shared disease architecture.

In this study, we apply these methods to both simulated and real phenotypes. Using simulations involving real genotypes, we find that LD between typed markers can significantly bias the 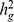 estimate and propose a correction to the GRM calculation, which we compare to a recently proposed approach [30]. In local analysis, we observe higher estimates of heritability with the adjusted variance-component strategy compared to traditional association and conditional analysis, particularly when the locus harbors multiple causal variants. Importantly, our LD residual correction ensures these statistics are not inflated under the range of disease architectures considered (unlike the correction of [30]). We estimate local 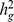 at known loci for nine common diseases finding a significant average increase vs. the variance explained by known associations, with individually significant increases for three of the traits. We also estimate local heritability at loci identified only in other related traits, showing significant enrichment in autoimmune disease for within-trait heritability at cross-trait loci. For RA, we analyze dense genotypes from 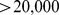 samples typed on the ImmunoChip data as part of the Rheumatoid Arthritis Consortium International (RACI). This significantly larger sample-size and deep genotyping empowers us to provide precise estimates on the significant increases in local heritability within RA and across non-RA autoimmune traits. Our results have important implications for fine-mapping study-design as well as the broader understanding of disease architecture and allelic heterogeneity.

## Results

### Overview of methods

Our fundamental goal is to explain as much of the local heritability as possible without upward bias. We consider four different estimators with unique individual properties: 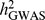, the variance explained by the single most associated SNP at a locus, computed directly from the effect-size of a univariate regression; 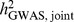, the variance explained by a conditional linear model of significant SNPs constructed by step-wise regression over all SNPs in the locus as described by [15]–[17]; 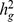, the heritability inferred with a standard variance-component constructed from all SNPs in the locus; and 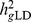, the heritability inferred with an LD-residual adjusted variance-component constructed from all SNPs in the locus. The LD adjustment is crucial in scenarios where LD patterns that are systematically different at causal variants can distort the observed sample relatedness and bias traditional estimates of 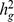, as previously demonstrated by [30]. Our proposed correction uses linear regression to transform each SNP into an “LD residual” of any correlated preceding markers and construct the GRM from these residuals. We compare this correction to LDAK, the re-weighing solution of [30], as well as other strategies (see Methods).

### Simulations using WTCCC genotype data

#### Impact of LD on genome-wide estimates of 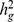


To be confident that our conclusions on excess local heritability are accurate, we first seek to get approximately unbiased estimates for genome-wide 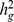 in simulations with realistic genetic architectures. Using the WTCCC1:CAD cohort (see Table S1), we sampled 5,000 of the genotyped SNPs to be causal variants such that a fraction 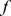 of the markers is low-frequency (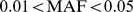) and generate a quantitative trait for which these markers explained 0.8 of the variance. We stress that in all instances the causal variants were always present in the GRM and that an unbiased 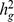 should equal the induced 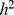 of 0.80. We then estimated the genome-wide heritability from genotyped or imputed SNPs using the standard GRM as well as four LD-adjustment strategies (see: Methods) over a range of 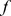 from 0 to 1 (Figure S1, ). We find that the standard estimate can be significantly biased in both directions depending on the disease architecture and typed markers (Table S2). For genotyped markers, the standard estimate tends to be inflated when causal variants are primarily common, and sharply deflated when the causal variants are primarily uncommon, dropping to as low as 0.50. This is likely caused by the fact that uncommon SNPs generally to have fewer other markers in high-LD and therefore become underrepresented in the GRM, deflating their contribution to heritability. With imputed markers, on the other hand, the standard estimate exhibits consistent deflation across all low-frequency variant cut-offs, dropping to 0.55 when all causal variants are uncommon. Examining the LD-correction schemes, we see that the LD-pruning strategy eliminates most of the bias but also reduces the measurement by roughly 25% due to the removal of correlated markers that offer some independent contribution to phenotype. The three non-pruning methods perform roughly similarly, all removing most of the bias without suffering from the deflation of LD-pruning. For genotyped SNPs, the regression-based LD residual had a slightly lower error (Table S4), while the LD shrink based on pairwise correlation resulted in highest error; with LDAK falling in the middle. For imputed SNPs, LDAK continuously outperforms the other methods, with LD residuals exhibiting a slight downwards bias. Since all three methods have multiple parameters it is likely that these differences are largely due to proper parameter tuning. However, one key advantage of the LD residual technique is that it does not exhibit statistically significant inflation under any disease architecture or platform tested, while both LDAK and the LD shrink can yield statistically significant upward bias in both genotyped and imputed SNPs (by z-test from mean and observed standard error over multiple simulations, after correcting for 11 tested frequency bins (Table S2, Figure S1). Likewise, the LD residual also yields a more conservative estimate of the standard error which, unlike the other methods, is never lower than the observed standard deviation of the estimate over all the simulated architectures (Figures S2). This conservative behavior is particularly important for our aim of placing an accurate lower bound on components of heritability.

To confirm that this deflation is caused by LD and not the allele frequency distribution, we permuted the carrier status of each marker and performed the above experiment again. This permutation procedure effectively removes any LD and results in independent genotypes as if sampled from the observed allele frequency spectrum. As shown in Figure S6, the inferred 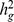 was never significantly different from the truth across all disease models. We note that when causal variants are sampled randomly from all typed SNPs the standard 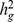 estimate is approximately unbiased and confirmed this in our simulations; in this scenario, the 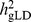 estimate exhibited a slight downward bias (95% of the true 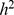 on average; Table S3), consistent with our previous findings that the estimate is conservative.

#### Performance of methods for local estimation of heritability

Having established a method for well-controlled estimates. We use real genotypes from the 7,923 WTCCC2-UC samples typed at 447,945 SNPs after QC (see Methods, Table S1) to simulate phenotypes from a range of disease architectures with each locus centered around 1–10 causal variants sampled from common or low frequency alleles (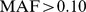 and 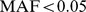 respectively) and normalized SNP effect-sizes drawn from the standard normal such that each SNP explains equal phenotypic variance in expectation (other distributions were also considered, see Methods). The simulated disease architecture mimics a large-scale GWAS and consists of 180 loci explaining a total trait heritability of 0.1, the number and 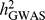 of loci recently identified in height [4] (see Methods). To quantify the upper-bound on heritability that can be explained with each method we first analyze simulated traits where all causal variants have been typed and are present in the set of analyzed SNPs. Table 1A,B shows the fraction of total heritability inferred from these simulations. For the standard unadjusted estimate local 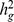, we see severe deflation with a single low-frequency causal variant (61% of the true heritability) and slight but statistically significant inflation with a single common causal variant (108% of the true heritability), with similar results for multiple causal variants. On the other hand, the adjusted estimates (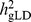) from these same simulated phenotypes are slightly conservative (down to 88% of the true heritability) but never exhibit severe deflation or significant inflation and, importantly, are consistent across all trait architectures. The consistently conservative behavior (avoiding upward bias in 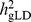) of our LD adjustment approach in extensive simulations that we conducted is a particularly attractive feature for our analysis as it ensures robust lower-bounds (modulo standard error) in real data regardless of disease architecture.

**Table 1:** Fraction of simulated local heritability explained in WTCCC2 genotypes.

	# Low-frequency typed causals:				
A:	1	2	3	5	10
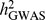	100%	82%	69%	55%	37%
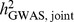	100%	85%	74%	59%	44%
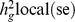	**61%** (1%)	**65%** (1%)	**61%** (1%)	**58%** (2%)	**60%** (1%)
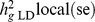	94% (2%)	92% (2%)	97% (3%)	95% (2%)	**90%** (2%)
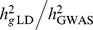	0.94	**1.13**	**1.40**	**1.71**	**2.37**

Analysis of simulated disease architecture with 180 causal 1Mbp loci yielding a true 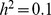. In each locus, 1–10 causal variants were sampled from either low-frequency (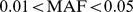) of common (MAF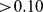) WTCCC2 SNPs. For each of four methods tested, the fraction of local heritability identified by the method is reported over 50 simulations (with standard error in parenthesis). Top two panels correspond to experiments with observed causal variants and bottom two panels to experiments with causal variants hidden. In A and B only (where causals are typed), bold-faced 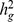 and 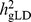 represents significant difference from 100% by z-score at 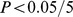 (accounting for 5 architectures tested). The ratio of 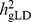 to 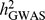 is reported in the bottom row of each panel (with bold-face indicating significance by t-test at 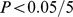).

Next, we consider these methods in the realistic scenario where causal variants are untyped, by removing the causal variant(s) from the set of SNPs analyzed. Our benchmark is 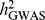, the variance explained by the single best tagging marker (see Methods). Table 1C,D shows that 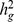 and 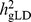 have relative results consistent with those observed over typed causal variants with a lower overall mean due to incomplete tagging. On the other hand, while the 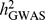 is roughly equal to 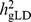 when one variant is causal, 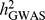 (unlike 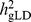) decreases greatly as the number of causal variants grows. As with the previous simulation, this decrease is not directly proportional to the number of causals because the GWAS SNP is always selected as the SNP with highest effect. The 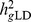 metric is always greater than or equal to 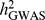, with as much as a 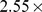 increase in variance explained when ten common causal variants are present. As before, we observe that the unadjusted 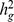 can be higher than 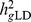 when causal variants are common. Since we generally do not know the underlying disease architecture and wish to avoid any upward bias, we prefer to use 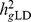. We also compare to the joint regression-based analysis and observe that while it can increase explained variance by as much as 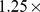 (in the ten low frequency causal variants scenario) it consistently recovers less of the true heritability than the variance component approach.

Analogous simulations over WTCCC1 data drawing causal variants from genotyped or imputed SNPs over several different disease architectures (including fewer loci and different effect-sizes) exhibited the same patterns (see Methods, Table S5,S6,S7,S8,S9). We also evaluated the impact of including imputed SNPs in the local heritability analysis, and found that while the absolute estimate increases (particularly for rare variants) the additional heritability recovered by 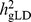 beyond 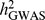 is lower, with the former exhibiting increased variance due to the substantially higher number of SNPs (Table S10,S11). Unlike our genome-wide estimates, no significant difference between LDAK and the LD-residual adjustment was observed in the local analysis (after accounting for five disease architectures tested) due to generally increased variance. Given the sporadic upward bias in LDAK in the genome-wide simulations, we focus on the LD-residual adjustment in real data but present results from both methods in the supplements.

Although our primary goal is to obtain the highest mean estimate without upward bias, we also examine the power to detect a statistically significant increase in local 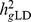 versus 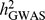. Specifically, we use the analytical standard error of the inferred 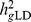 in each of the simulations scenarios from Table 1 to report the fraction of 100 random simulations where 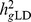 is significantly higher than 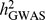 (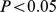; see Methods). This power computation strongly depends on the sample-size, total 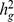, disease architecture and average relatedness of the samples, and may therefore vary across different datasets. In Table S12, we observe that the power to detect a statistically significant increase in 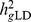 versus 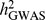 is highly variable, ranging from below 10% for the 1 and 2 causal variant models to as high as 56% for the 10 causal variant model. When we perform the same analysis in all 15,000 WTCCC1 samples (Table S13) we see that the power is very high, averaging 47% and reaching nearly 100% for the three causal variant architecture. These findings indicate that in very large studies our approach can conclusively identify additional local heritability.

These simulations demonstrate the value of the variance-component approach in recovering true additional heritability beyond that explained by individually or jointly significant markers, especially in the presence of multiple causal variants. The same simulations were also performed on the ImmunoChip genotypes, with similar trends described in more detail below.

### Genome-wide heritability analysis of WTCCC case-control traits

**Figure 1:**
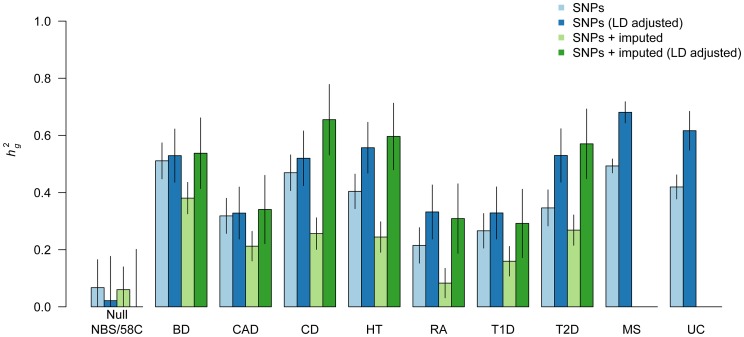
Heritability of genome-wide SNPs for nine complex traits. Components of heritability for typed markers (blue) over nine traits and imputed markers (green) over seven WTCCC1 traits shown. Light bars correspond to estimates from the standard variance-component and dark bars correspond to estimate from LD-adjusted variance-component. Two control sub-groups (NBS and 58C) tested against each other as negative control; diseases tested are Bipolar Disorder (BD), Coronary Artery Disease (CAD), Crohn's Disease (CD), Hypertension (HT), Rheumatoid Arthritis (RA), Type 1 Diabetes (T1D), Type 2 Diabetes (T2D), Multiple Sclerosis (MS), Ulcerative Colitis (UC). Autoimmune traits (CD, RA, T1D, UC, and MS) excluded the well-studied MHC region. All traits exhibit an increase after LD adjustment, indicative of a genetic architecture that is shifted towards low-frequency causal variants. Error bars show analytical standard error of estimate.

We first analyze the genome-wide heritability explained by genotyped SNPs in the nine WTCCC1 and WTCCC2 traits (Table S1). Figure 1 shows the results of this analysis for unadjusted and LD-adjusted estimates performed over genotyped and genotyped+imputed SNPs (2.1 million 1000 Genomes [31] SNPs on average; see Table S1) separately. Results are shown on the observed scale. (Results on the liability scale are provided in Figure S4; all numerical values are provided in Table S14,S15.) We note that stringent quality-control is imperative for heritability analysis, where many small artifacts can compound into significant inflation of the genome-wide estimate [23]; this effect can be exacerbated by LD-adjustment methods, which will tend to promote low frequency variants that may be especially prone to QC issues. As in other studies [23], [30], we use a series of highly conservative QC filters to stem this problem, at the cost of filtering out many potentially informative markers (see Methods). The absence of any significant false heritability between the two control cohorts, particularly after LD-adjustment, indicates that genotyping artifacts are unlikely to be substantial (Figure 1). We note that in the presence of strong artifacts [30], propose an elegant solution of estimating SNP weighing scores from an independent population, and a similar strategy can be applied to the LD-residual adjustment.

For all traits we see that the LD-adjusted estimate from typed SNPs is higher than the corresponding unadjusted estimate, with an average of 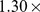 for genotyped SNPs. Previous work has shown the standard estimate to be robust when the trait is infinitesimal, i.e. where all SNPs are causal with normally distributed effect-sizes [32], [33]. However, as demonstrated in our simulations and in [30], non-infinitesimal traits with systematically less LD between rare and low-frequency variants will underrepresent those variants in the un-adjusted kinship, resulting in deflated 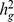 estimates when a majority of the causal variants are low-frequency (Figure S1). The increase in adjusted estimates on real data therefore implies a genome-wide genetic architecture for these traits that is generally shifted towards low-frequency variants. As in our simulations, the effect of LD-adjustment is even stronger when imputed SNPs are included (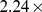 more on average, comparing dark-green to light-green bars), demonstrating the downwards bias introduced by an abundance of imputed markers without LD adjustment. Indeed, without adjustment, all of the traits exhibit lower 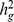 after imputation. Interestingly, even though imputation increases the total number of markers by 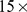, the adjusted estimate from imputed SNPs is, on average, only 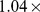 higher than the corresponding estimate from genotyped SNPs. Because the LD adjustment effectively removes any new SNP that is a linear combination of nearby SNPs, this would be consistent with imputation providing information similar to such linear combinations [34]. This is further supported by the fact that the sum of LD-adjusted SNP variances (roughly corresponding to the independent number of SNPs) for imputed SNPs was only 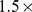 higher than that of typed SNPs. These findings do not minimize the utility of imputation for mapping, where individual effect sizes are important, but does imply that imputed variants are not explaining dramatically more missing heritability. Based on these findings and our previous simulations with imputed variants, we restrict our subsequent variance-components analysis to the genotyped data only.

### Local heritability analysis of WTCCC traits at known GWAS loci

**Figure 2:**
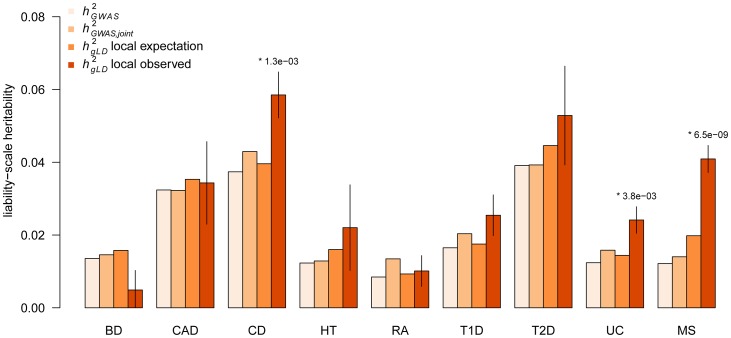
Local heritability around known GWAS loci. Components of heritability inferred at previously known GWAS loci. 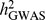 computed from leading SNP effect-size; 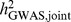 computed from joint model of all known and significant SNPs in region; local expectation computed from 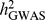 and fraction of genome analyzed; and 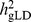 computed from LD adjusted variance component over all loci. (*) indicates statistically significant increase over expectation after accounting for nine tests. Error bars show analytical standard error of estimate. Autoimmune traits (CD, RA, T1D, UC, and MS) excluded the well-studied MHC region.

Next, we infer the amount of local 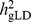 around the GWAS loci for the nine traits and compare to the corresponding 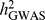 and 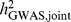 values (Figure 2, Table S16). When computing the increase in 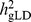 (and its statistical significance), we always account for 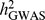 and the local expectation, i.e. the increase that would be expected by chance based on the total genome-wide 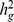 and the fraction of genome covered by the variance-component (see Methods). Across all the nine traits we find a consistent excess of local heritability, with an average increase of 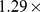 over the local expectation (combined 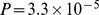). These results were consistent with the LDAK-based adjustment, which had a mean increase of 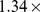 (Table S18). P-values were computed using a z-test and consistent to different definitions of 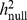 (see Methods), but an analysis involving a comparison to random regions of the genome also produced similar results (see Methods, Table S17, Figure S5). Three of these traits (CD, UC, and MS) show individually significant increases (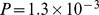; 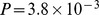; and 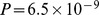 respectively). The regression-based analysis of jointly significant markers (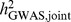) yields an average of 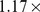 more heritability than 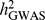. In instances where there are multiple known associations at a locus, only the leading SNP is included in 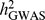 but all of the known associated SNPs are automatically included in 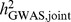, demonstrating that previously known locus heterogeneity still does not explain as much heritability as the 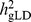 estimate. On average, these loci are explaining 11% of the genome-wide 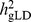 with 1.1% of the genome. Interestingly, the 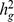 estimate with no LD-adjustment also yields increased local heritability for all phenotypes with an even higher average increase (Table S18). Given that our simulations show an increase in unadjusted estimates only when the underlying causal variant is common (Table 1), this increase in real data suggests that most causal variation in these GWAS loci originates from common causal variants (in contrast to the rest of the genome; see above).

The presence of significant additional heritability in individual traits raises the question of whether it is coming from a single poorly-tagged causal variant or multiple independent causal variants. In our previous simulations, an increase in local heritability is not expected under the single causal-variant model and the ratio of 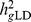 to 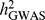 has a direct relationship to the number of causal variants. For the WTCCC2 data, a single rare or common untyped causal variant is expected to yield an 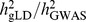 of 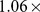 and 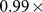, respectively (Table 1 C,D). Both are lower than our observed average of 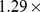 in real data, and much lower than significant increases of 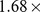 and 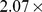 in UC and MS (Table S16). These results are therefore unlikely to arise simply due to all loci harboring a single poorly-tagged causal variant, with the point estimate of 1.29 indicating a likely architecture of 2–3 causal variants at the average locus. However, we caution that the variance of this ratio observed in simulations is very high (for example, 18% of the single common causal simulations have a local increase greater than 1.29), making it difficult to reject the single-causal variant hypothesis at this sample-size. From our previous power estimates (Table S13), we observe that at a sample-size of 15,000 power to detect multiple causal variants approaches 100%, allowing us to distinguish between these two scenarios.

We note that some of the GWAS loci we analyzed were genome-wide significant in the WTCCC data and could potentially exhibit inflated effect-sizes due to winner's curse if discovered in this cohort. However, because the heritability from variance-components and GWAS SNPs are inferred in the same data, we expect any effect-size inflation to impact both estimates equally, making our relative comparisons robust even in the presence of biases. In light of this and the small fraction of such loci actually present (8% averaged over the 7 WTCCC1 traits) we do not believe winner's curse to have had an impact on these results.

### Local heritability analysis of WTCCC traits at GWAS loci associated to related traits

Recent analyses of multiple phenotypes have demonstrated significant correlations in genetic architecture for certain groups of related traits [23], [24], [35], [36]. Unique to the local variance-components approach, we can also compute components of heritability at known GWAS loci from multiple related traits without having genotypes for those traits. This measure provides an estimate of the additional variation that would be explained by fine-mapping loci associated with one trait within the affected samples of another; for example, analyzing known Ulcerative Colitis loci in a study of Crohn's Disease. We expect this to be informative when the traits have correlated genetic architectures, with causal variants that only reached statistical significance in one trait potentially explaining heritability in the other. One example of such related traits is the class of autoimmune disorders, which are known to have a shared disease architecture as well as many instances of overlapping GWAS loci [22], [37]–[40]. For each of the nine traits, we consider the amount of heritability explained by loci that were previously associated to one or more other autoimmune diseases but not to the focal trait. By definition, the 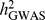 for these loci is zero, and so we compare to the local expectation, i.e. what would be expected by chance from the genome-wide 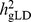 and locus size (see Methods). As with all other analyses, we specifically exclude the MHC for all autoimmune diseases so as to investigate the patterns of shared heritability outside of this well-studied region.

**Figure 3:**
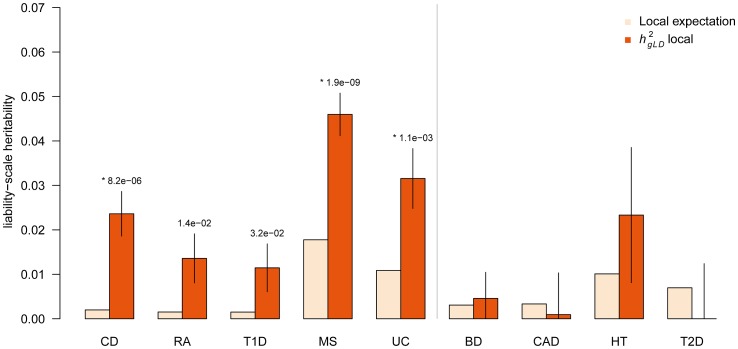
Heritability of known autoimmune disease loci. Components of heritability inferred at previously known autoimmune trait loci not identified for focal trait. Local expectation computed based on fraction of genome analyzed. (*) indicates statistically significant increase over expectation after accounting for nine tests, respectively. Error bars show analytical standard error of estimate. All analyzed autoimmune traits (Crohn's Disease, Rheumatoid Arthritis, Type 1 Diabetes, Multiple Sclerosis, and Ulcerative Colitis) all exhibit significant increase in local 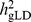 where non-autoimmune traits (Bipolar Disorder, Coronary Artery Disease, Hypertension, Type 2 Diabetes) exhibit no significant increase. Autoimmune traits excluded the well-studied MHC region.

Figure 3 (numerical results in Table S19,S20) shows the results of this analysis, as well as the increase in heritability explained compared to the local expectation. The five autoimmune traits have the highest relative increases and are unique in being statistically significant. On average, the loci in the autoimmune traits explain 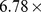 more heritability than the local expectation (combined 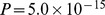), compared to 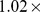 more for the non-autoimmune traits (combined 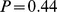). Both results were consistent with the LDAK-adjusted estimate of 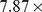 and 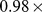 respectively (Table S20). We again confirmed all significant z-test results using an empirical expectation by sampling random regions of the genome (see Methods, Table S17, Figure S5). Importantly, these results were not substantially different after accounting for increased heritability in coding regions, with the average increase after correction still significant at 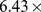 (see Methods, Table S21). We stress that these estimates specifically exclude any known loci for the respective disease; for example, the results from RA represent analysis of known autoimmune disease loci not identified in RA, and likewise for all of the other traits. As such, the additional heritability we identify would not have been found in a traditional targeted fine-mapping study that focuses only on trait-specific loci. Combining these results with the trait-specific analysis, we observe an average of 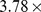 more 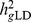 than 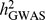 at the union of autoimmune and disease-specific loci, individually significant across all the autoimmune traits (Table S22). On average, these loci are explaining 27% of the genome-wide 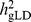. Most significant are the increases for MS and CD, with 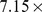 (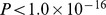) and 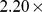 (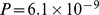) more local 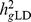, respectively.

Overall, we find that the class of autoimmune traits has a shared genetic architecture at known GWAS loci that can be leveraged to explain significant additional heritability. Loci found in one autoimmune trait are expected to harbor significantly more 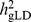 for other traits (beyond what is expected from lying near coding regions) and can therefore be important targets for fine-mapping analysis.

### Heritability analysis of Rheumatoid Arthritis in ImmunoChip cohort

We estimate components of local heritability for Rheumatoid Arthritis in 23,092 samples of European origin typed on the ImmunoChip platform, recently analyzed for association by Eyre et al. [41]. The increased SNP density of this data is expected to provide higher power for local heritability analyses, and we again compare 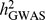, 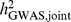, 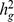, and 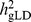 using simulated phenotypes from ImmunoChip genotypes (see Methods). We again observe an inflated 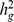 and un-inflated 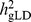, though the latter is more conservative than in previous simulations (Table S23). Overall, the higher density ImmunoChip results in a greater expected increase when considering all SNPs, particularly when variants are low-frequency.

We now consider real RA phenotypes. Of the 13 RA GWAS loci analyzed in the WTCCC1 data, 10 are also present on the ImmunoChip and we re-estimate local 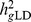 at this subset of 10 loci in both studies for comparison (Table 2A). The ImmunoChip data exhibits an increase in additional heritability explained over local expectation of 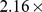 (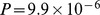), compared to 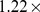 (non-significant at 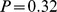) in the corresponding WTCCC1 loci. The ImmunoChip also exhibits a significant increase in heritability explained compared to 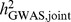 and local expectation, with an increase of 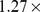 (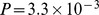). The ImmunoChip also contains 17 of the 24 non-RA autoimmune disease loci, also allowing us to perform the analysis of non-RA autoimmune loci. Again, we observe the local heritability to increase between the WTCCC1 and ImmunoChip data from 0.012 to 0.018, with the latter resulting in an increase of 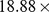 compared to local expectation (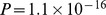, Table 2B). Examining all relevant loci on the ImmunoChip, which are more likely to come from studies performed after the WTCCC, both local increases were lower but more significant due to the additional data analyzed.

**Table 2:** Replication of heritability of Rheumatoid Arthritis in ImmunoChip data.

A: RA GWAS Loci							
Cohort (# Samples)	Loci (# Loci)	% genome	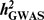	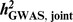	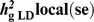	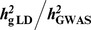	P-Value
WTCCC (4,395)	Affy (13)	0.5%	0.008	0.013	0.010 (0.004)	1.09	4.2×10^−01^
WTCCC (4,395)	Affy 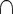 IChip (10)	0.3%	0.007	0.010	0.010 (0.004)	1.22	3.2×10^−01^
RACI (23,092)	Affy 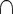 IChip (10)	0.3%	0.006	0.009	0.014 (0.002)	2.16	9.9×10^−06^
RACI (23,092)	IChip (29)	0.9%	0.027	0.033	0.042 (0.003)	1.57	6.7×10^−08^

Components of heritability for Rheumatoid Arthritis are shown at (A) previously known GWAS loci and (B) non-RA autoimmune loci in 4,395 WTCCC samples and 23,092 ImmunoChip samples. Autoimmune loci specifically exclude known RA regions, yielding 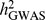 equal to zero. Increase shown for 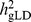 over 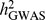 and local expectation from fraction of genome covered, highly significant for ImmunoChip samples in both panels. The well-studied MHC region was excluded entirely.

For consistency, we have assumed the same total 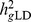 of 0.14 in both of the data-sets when computing the local heritability expected by chance, though this is likely an underestimate for the dense typing on the ImmunoChip. Likewise, the densely typed ImmunoChip sites also tag some markers outside of the variance-component region, effectively increasing the local expectation. Using 1,000 Genomes data, we find that a sequenced variant within 500 kbp of the studied regions is tagged with an average 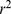 of 0.33 by the ImmunoChip sites in these loci, so we also consider a local expectation where each region is increased by 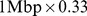 of “flanking” length. However, irrespective of whether we use a total 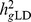 of 0.40 (the total 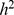 estimated in previous studies excluding MHC [42]) and/or include the flanking regions, the local heritability identified at these loci remains strongly significant (Table S24). Overall, the ImmunoChip data shows local 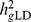 for RA at 27 known (RA+other) autoimmune loci to be 0.032, 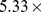 higher than that explained by the individual RA GWAS SNPs (0.006) and 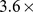 higher than the joint GWAS model (0.009).

The variance-component method allows us to estimate local 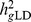 at regions that are suggestive of harboring a secondary signal in this data. Specifically, Eyre et al. [41] analyzed these samples for conditional association and identified six loci that had a significant secondary signal. Predictably, when we restrict our analysis to these loci we confirm that the joint model increases heritability by 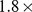 over the associated SNP, but we also find the local 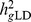 to be even higher with a 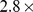 increase over the associated SNP and highly significant compared to local expectation (Table S25). Though the joint analysis has high power in this large cohort, the variance-components model still reveals additional hidden heritability. Similarly, Diogo et al. [43] fine-mapped 25 known RA loci and searched for the presence of secondary associations driven by variants in the protein-coding sequence of biological candidate genes, identifying strong enrichment of association at 10 coding variants (9 loci) but no individually significant variant. We examine these 9 loci in the ImmunoChip data and again observe an increase in heritability from the joint analysis of 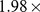 compared to the leading SNPs, but an even higher increase in local 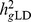 of 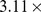 which is more significant at 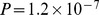 than the permutation-based 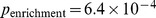 reported by Diogo et al. (Table S25).

Overall, the higher density and sample-size of the ImmunoChip data empowers us to identify the presence of significant additional 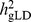 at known RA loci as well as known non-RA autoimmune loci, beyond the heritability explained by standard mapping approaches analyzing the same data.

## Discussion

In this work we have sought to explain additional heritability at known GWAS loci by using large-sample SNP data. Specifically, we have utilized variance-components models that estimate the total contribution of all typed markers in the sample and do not require individual markers to be genome-wide significant. In applying these methods we have quantified biases in the standard 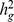 estimate when the underlying disease architecture is non-infinitesimal and LD is systematically different at causal variants (as recently identified by [30]). To address this, we have proposed and compared several methods that seek to adjust the covariance matrix such that this correlation between markers is accounted for. In particular, we find the method of using LD residuals in computing the kinship to provide accurate estimates with no observed upward bias, in contrast to the proposed LDAK strategy [30] which yielded upward bias in our genome-wide simulations (though it exhibited lower mean error in imputed data). We thus recommend that the LD-residual approach be used in preference to LDAK when one is seeking lower bounds on the estimate of 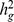, as we are here.

Applying the LD-residual to known GWAS loci for nine WTCCC1 and WTCCC2 traits, we see that LD-adjusted estimates are nearly always higher than the unadjusted estimates, suggesting that the disease architecture is indeed shifted towards low-frequency variants for most traits. Understanding this phenomenon and applying and LD-adjustment method is therefore important for accurate estimation of 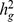 in future studies. An alternative framework is the Bayesian sparse linear mixed model, which attempts to infer the underlying genetic architecture jointly with the 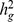 and can provide more accurate estimates under certain disease architectures but requires significant computational resources (e.g. running time of 77 hours for a data set with 3,925 samples) [44].

Looking at previously known GWAS loci, we showed by simulation that the LD-residual adjusted variance-components approach is not inflated and can uncover additional heritability beyond that observed by the leading tag SNP, particularly when there are multiple underlying causal variants or tags. In analysis of nine dichotomous traits, we find a significant average increase in heritability explained of 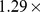 (combined 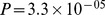), with three traits exhibiting individually significant increases consistent with the presence of multiple causal variants on average. The latter finding is supported by previous work showing that loci with a single causal variant are unlikely to explain substantially more heritability then the GWAS SNP and hypothesizing multiple underlying causal variants [8]. However, though our simulations show that increased heritability is an indicator of multiple causal variants on average, the current sample size is not sufficient to reject the possibility that this local increase is caused by a single causal variant being poorly tagged by the leading GWAS SNP. We extrapolate that as sample sizes reach the tens of thousands our method can conclusively draw distinctions between these two scenarios.

Because the LD-unadjusted method tends to be deflated when the underlying causal variant is low-frequency (Table 1), we can use the unadjusted estimate as an indicator of the causal allele frequency. The fact that all but one of these traits exhibit an unadjusted local 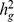 that is higher than the 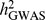 strongly suggests that the bulk of causal variation at these known loci does not lie in low-frequency variants. This is consistent with the recent findings of Hunt et al. [45] in a large-scale sequencing study that demonstrated minimal rare-variant heritability for 25 known auto-immune disease risk genes. This is in contrast to our genome-wide analysis that yielded additional heritability after LD-adjustment, indicative of a shift toward low-frequency markers. Taken together, we hypothesize that the causal frequency spectrum at these known loci is substantially different from that of the rest of the genome. In light of this finding, we caution against extrapolating the genome-wide disease architecture from known GWAS loci, as done in Hunt et al. and other studies [45]–[48].

We also applied this technique to loci that have been discovered in related traits but not in the focal trait. Additional variation would be found in instances where causal loci are shared across multiple traits but have only been mapped in one trait, allowing us to estimate the efficacy of a fine-mapping study design incorporating these loci. For autoimmune diseases we see a significant amount of excess heritability at such related-trait loci with an average of 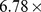 more than expected by chance. Relative to the known 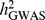, the greatest increase from the union of trait-specific and related-trait loci is observed in MS (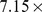) and CD (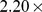). This finding is substantiated by the fact that non-autoimmune traits exhibit no such significant increase and serve as negative controls. Where previous studies have documented overlap between causal variants from autoimmune disease [22], [40], we show that this is a wide-spread phenomenon expected to account for an average of 27% of total 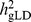 over five auto-immune traits. Our analysis is complementary to recent methods that construct multivariate variance-components models which directly estimate the genetic correlation between multiple traits [23], [24]. In contrast to those studies, our approach requires only the genetic information from a single trait of interest, allowing us to analyze components of heritability between many autoimmune traits without having their genetic data. Looking forward, this strategy can be used to analyze other classes of related phenotypes such as metabolic traits [24] and psychiatric disorders [36]. Given that we observe GWAS loci to have fundamentally different disease architectures from the rest of the genome, our method will still not capture the genome-wide correlation between the two traits. A potential future application is local heritability analysis with the multivariate variance-components model, merging these two strategies.

For RA, we repeated our analysis in a much larger cohort typed on the ImmunoChip and found significant additional heritability. Where the GWAS analysis of this data by Eyre et al. [41] found 6/45 loci containing a secondary marker, we quantify the overall amount of additional heritability to be 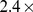 than 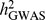. While Eyre et al. identified a significant correlation between their associated loci and genes with auto-immune function, we additionally observe 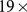 more heritability than expected by chance in non-RA auto-immune loci (Table 2), a highly significant increase. These findings demonstrate the effectiveness of our method in quantifying components of heritability from high-density data. Loci from the other traits we examined have also recently been analyzed large fine-mapping studies. Jostins et al. [40] found that 30/163 loci associated with Crohn's Disease or Ulcerative Colitis exhibit significant secondary effects, and all loci have an 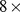 higher chance of being associated with immune-function genes. Likewise, we observe significant local and related-trait heritability for Crohn's Disease. On the other hand, Shea et al. [25] re-sequenced one locus for T2D and Maller et al. [20] densely genotyped 11 loci for CAD and T2D, with neither study identifying significantly more heritability. This too is consistent with our failure to observe significant increases in heritability for these traits, though both sets of negative results may be due to the small number of loci and samples examined.

Two recent publications by Ehret et al. and Ke [16], [17] propose methods to quantify the amount of recoverable heritability at known loci by selecting a conditional linear model. The conceptual distinction between these methods and our approach is that they explicitly focus on a pruned and p-value restricted set of markers and are therefore limited by power to detect association within the analyzed sample. The Ke strategy differs from that of Ehret et al. in the specific threshold values and that it does not depend on an external set of samples for estimating unbiased effects; as such, it is likely to be the less conservative estimate of local heritability and the one we selected for comparison. Because these strategies only focus on loci where conditionally nominal SNPs are present, they do not provide a complete analysis of all known loci together. While it is possible to incorporate many more SNPs into a complex multiple regression and estimate the total fraction of phenotypic variance explained, this estimate will be highly biased proportional to the effective number of SNPs divided by the effective number of samples, a difficult ratio to quantify in the presence of LD between SNPs and sample structure. On the other hand, the local variance-components model provides an approximately unbiased estimate of the total heritability explained by all SNPs, allowing us estimate components from putative loci without significant associations, as we do here with related traits. Both in simulations and in real data, we find that our strategy identifies more additional variation than the standard linear model.

One limitation of the current variance-components strategy is that analysis of ascertained case-control traits can lead to underestimates of 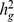 when the ratio of SNPs to samples is low (A.L.P., unpublished data), as can be the case when analyzing a small number of loci. This would lower the power to detect significant additional heritability and yield local estimates that are a conservative lower bound. Quantifying and correcting for this phenomena in case-control traits is an important area of future study. Other future directions for this work include the estimation of local heritability over more complex annotations of putative regions [49] as well as the use of local heritability for mapping previously unknown loci akin to group-wise tests [50], [51].

The torrent of large-scale sequencing studies will do much to inform our understanding of the genetic architecture of common diseases, but the design of such studies also motivates the inference of disease architecture from currently available data. The strategies outlined here demonstrate a great diversity of allelic heterogeneity within and between traits, informing our assumptions for future GWAS and fine-mapping analysis.

## Methods

### Data

We examined data from the Wellcome Trust Case Control Consortium (WTCCC) versions 1 and 2. These datasets have been outlined in [13] and [12], [40], and we provide summary details in Table S1. Unlike GWAS studies, heritability estimates can be particularly sensitive to individually small artifacts/batch-effects [52], which can add up over many SNPs to exhibit false heritability [29]. To account for this, we apply several additional layers of quality control.

We also examined 23,092 samples of European origin typed on the ImmunoChip platform (32% cases for Rhematoid Arthritis), recently analyzed for association by Eyre et al. [41]. For this data, we followed the QC protocol of Eyre et al. [41] and also excluded any SNPs below 1% allele frequency.

#### WTCCC quality control

For all analysis of real phenotypes, we performed rigorous quality control to account for genotyping error. For each cohort, we removed any SNPs that were below 0.01 minor allele frequency, above 0.002 missingness, and had deviation from Hardy-Weinberg equilibrium at a p-value below 0.01. Then, for each case-control cohort we removed SNPs that had differential missingness with p-value below 0.05. The entire procedure retained approximately 140,000 markers in each WTCCC1 case-control cohort; 450,000 markers in the WTCCC2-UC; and 400,000 in the WTCCC2-MS (Table S1).

Genetic structure due to ancestry has been shown to introduce subtle biases into 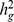 estimates. This is a particularly serious problem for local estimates, where ancestry can explain additional variation in phenotype from other parts of the genome. To account for this, we excluded one of any pair of samples with normalized SNP covariance 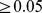 and carried out principal components analysis to identify the 20 most significant eigenvectors within the QC genotypes. We subsequently performed five rounds of outlier removal whereby all individuals more than 6 standard deviations away from the mean along any of the top 20 eigenvectors were removed and all eigenvectors recomputed. The entire QC process retained approximately 4,300 samples in each WTCCC1 case-cohort; 8,000 samples in the WTCCC2-UC cohort; and 15,000 samples in the WTCCC2-MS.

For all autoimmune diseases analyzed (RA, CD, T1D, UC, MS) we also exclude from the analysis any SNPs in the region around the MHC locus (chr6:26–34 Mbp), which has been repeatedly documented to have a complex LD structure and many heterogeneous variants of strong effect for these traits. Heritability of genotyped SNPs for BD, CD, and T1D (with/without the MHC) have previously been reported [29].

#### Imputation

After performing quality control on the WTCCC1 samples, we also performed imputation from the 1,000 Genomes Project [31] reference panels (Integrated Phase 1 v3). A total of 36,648,992 SNPs in all 1,092 samples from all reference populations were analyzed together. All WTCCC1 samples were analyzed together, with each chromosome first pre-phased using the HAPI-UR algorithm [53] (see Web Resources) with standard parameters and three rounds of phase inference followed consensus voting. Next, we ran IMPUTE2 [54] on the pre-phased data in windows of approximately 1 Mbp and default parameters. The full panels were used as a reference and only those imputed markers with an IMPUTE2 information metric higher than 0.6 were retained, for a total of 8.2 million imputed and genotyped SNPs. Finally, the same QC thresholds as those used for genotypes were applied on the imputed data except the maximum locus missingness threshold was increased to 0.05 as we expect batch effects to have less impact in this post-QC data. The final set of genotypes contained approximately 2.17 million imputed and genotyped SNPs per cohort. Due to the larger sample size and SNP density of the WTCCC2 data, we did not perform imputation for the UC and MS cohorts.

### Estimating heritability of typed SNPs

#### Variance components estimation

The variance-components method has previously been described in [33], and we summarize it here. Formally, we assume the phenotype is generated from a model 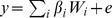 where 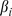 and 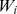 are the effect-size and genotype coding of SNP 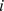, and 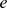 is environmental noise. Given a kinship matrix that relates all pairs of individuals, the phenotype variance is then defined as 
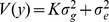
 where, assuming all of the SNPs have been rescaled/normalized to have equal mean and variance, 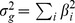 and the narrow-sense heritability 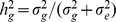. While the kinship matrix 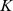 ideally represents the exact sample covariance over all causal variants, these values can be partially estimated directly from high-density SNP panels by computing the genotypic relatedness matrix (GRM) as 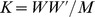 over the 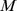 normalized SNPs in 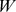. The variance-components can then be inferred using likelihood maximization under the assumption that SNP effects arise from the multivariate Normal distribution.

Variance explained by the GRM is estimated jointly with a residual component (the identity matrix) using restricted maximum likelihood (REML) [55], which properly accounts for the fixed-effects in the likelihood function. The Average Information coefficients [56] together with the first derivatives of the log likelihood function with respect to each variance component are used to iteratively converge on the corresponding heritability estimates. The inverse of the final Average Information matrix yields an estimate of the corresponding covariance matrix of the variance-component estimates [57] which is used to convert the variance-component estimates into 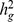 (by the Delta Method) as well as obtain the corresponding standard error of the 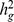 and 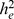 values (referred to here as the “analytical” standard error). In practice, a single affine-term (vectors of 1 s) and the top 20 principal components were also included as fixed-effects to account for population structure in all local and global estimates of heritability from real phenotypes (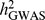, 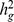, 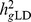, and 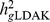). The estimation was performed using the GCTA software [58] (see Web Resources).

#### Liability-scale transformation for ascertained traits

Our analysis focuses on case-control traits with non-random ascertainment which makes it difficult to compare observed-scale heritability estimates across diseases or with other studies. To mitigate this, we assume the classical liability-threshold model [59] and also report all of our findings transformed to the liability scale. This transformation uses the proportion of cases in the sample 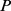 and the proportion of cases in the population, or prevalence 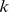 to transform an observed 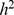 value to liability-scale:
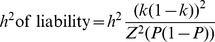
where 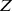 is the height of the standard normal probability density function at the threshold that truncates the proportion 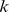 [32], [59]. The standard error of the estimate can be transformed accordingly. Because this transformation to heritability of liability is linear, any ratios and p-values we report for the transformed estimates are unaffected. We note that the accuracy of the transformed value depends on the level of trait ascertainment as well as the degree of relatedness in the cohort. [29] demonstrated the the transformation is robust in the WTCCC1 traits and we have also explicitly pruned the samples for related individuals, so we do not expect errors in the transformation to effect our results but care should be taken in applying this method to highly-ascertained or related cohorts.

### Accounting for non-uniform LD

The variance-components model assumes an idealized infinitesimal genetic architecture where every marker is causal and effect-sizes are normally distributed over the normalized variants. [33] showed that the model remains unbiased when causal variants are randomly sampled from the typed SNPs (though the analytical standard error on the estimate does exhibit bias as the number of causal variants becomes very low [30]). However, as demonstrated in [33], when causal variants are not randomly drawn from the typed SNPs, LD between markers can lead to over-representation of certain SNPs in the sample GRM and distort the estimated relationships between individuals, thereby distorting the final estimate of SNP-heritability. We describe and evaluate several methods that account for correlations between markers when constructing a GRM. In all cases, the goal is to reweigh or transform each SNP so that it is equally represented in a new adjusted genotype matrix. We caution that our simulations do not explore the robustness of this model in the presence of very rare variants (e.g. whole-genome sequence) where assumptions of normality may be strongly violated.

#### LD-pruning

One of each pair of markers that surpasses a 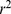 threshold is removed from the analysis. Formally, a sliding window is moved across the genotype matrix and the marker with the highest number of pairs over the threshold is removed greedily until no such markers exist. The GRM is then computed in the standard way over the remaining SNPs. Other estimates of heritability have been previously performed with 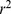 thresholds in the range of 0.1–0.3 [16], [42] and so we use 0.3 in our analysis. Lower thresholds are more likely to address the LD-bias, but will also lose more heritability due to SNPs with non-redundant information being excluded.

#### Transformation by linear regression (LD-residual)

Following the strategy proposed in [60], a new genotype matrix 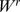 is generated where each marker is regressed onto the 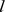 markers preceding it and transformed into the residual:
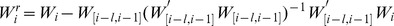
Each new genotype is then independent of the linear combination of preceding markers. If we consider the simple case of two markers that are highly correlated, this procedure will shrink the second marker to be the residual of the first with variance equal to one minus their squared correlation, effectively removing the redundant contribution from the analysis.

It's important to note that 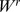 does not maintain the standard properties where each marker has 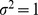, therefore the resulting GRM 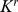 must be normalized by sum of the empirical variance:
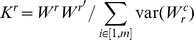
We use a SNP window that corresponds to the preceding 100 kbp (500 kbp for imputed data) and (arbitrarily) remove one of any pair of SNPs that have an 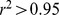 so that the relevant matrix inversions can be performed. We refer to estimates of heritability from the LD-residual matrix as 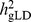.

#### Reweighting by pairwise correlation (LD-shrink)

Following the method described by [61] for population structure, we re-weight each marker according to the number of neighboring markers in high 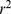. Formally, a weight is computed across the 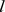 nearest markers:
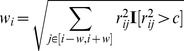
where 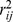 is the Pearson squared-correlation between genotypes 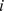 and 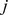; and 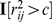 is a zero/one indicator for when the correlation surpasses threshold 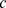. The markers are then re-weighted to form a new genotype matrix 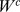, where the columns 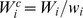. A final GRM is then computed and normalized by the sum of individual weights:
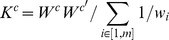
We set an 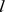 of 150 SNPs and 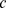 cut-off of 0.2 as suggested by [61].

#### LDAK

Recently, the impact of LD on heritability estimates was also quantified by Speed and colleagues [30], who propose a method for reweighing markers to account for LD. Their method, LDAK, examines the local SNP correlation matrix and computes optimal SNP weights by solving a linear program. This re-weighing can be thought of as an optimal variant of the Zou et. al. approach that also accounts for SNP distance. Both of these strategies are fundamentally different from our regression approach in that they only adjust the SNP weights rather than the SNPs themselves. We apply the LDAK 1.4 algorithm with default parameters (500 predictors for array, 1000 predictors for imputation). We refer to estimates of heritability from the LD-residual matrix as 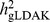.

Averaging over 10 runs, chromosome 1 of the WTCCC data (roughly 10% of the genome) was processed by LDAK in 1271 seconds, requiring 1426MB of memory; the LD-residual analysis implemented in EIGENSOFT (see Web Resources) took 1181 seconds, requiring 676MB of memory.

### Analysis of known GWAS loci

#### Standard GWAS analysis

For each trait, we identified known associated SNPs from the NCBI published GWAS catalog (version 2013-03-06). Any marker that is present in our typed or imputed data (after QC) defines a locus in the linear model and variance-component. We then include all known GWAS loci together in a linear model and compute the 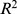 or variance-explained by the model. As in [16], we shrink the estimate by subtracting 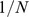 for each of the SNPs included (where 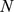 is the number of samples) and then transform to the liability scale. For the loci where multiple variants are present within a single megabase, we only include the single most associated SNP in this data (all SNPs are considered in the joint/conditional analysis described below). All SNP counts and allele frequency distributions for GWAS loci are detailed in Table S26.

#### Joint/conditional analysis

We follow the procedure outlined by Ke [17] for step-wise construction of a linear model which attempts to explain conditionally independent sources of association. Specifically, we include all known GWAS markers as initial predictors and all other nearby SNPs in the initial pool of 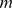 total putative predictors. We perform a standard univariate association and remove any markers that do not surpass a p-value of 0.05 corrected for 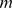 tests. We then iteratively add the conditionally most associated SNP to the model until no marker is conditionally significant at 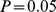. For loci where no marker is significant, we therefore only include the known GWAS markers (though a strict adherence to the Ke procedure would entirely exclude such loci and decrease the overall estimate). The final measure of 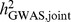 is then the 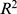 or variance-explained of the final model; shrunk by subtracting 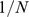 for each of the predictors in the final model (where 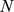 is the total number of samples); and transformed to the liability scale. This procedure is similar to the model selection in [15] but we require SNPs to be conditionally significant at 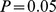 rather than at 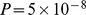, making this estimate much less conservative than the one in [15] (ignoring differences due to meta-analysis).

#### Estimating components of local heritability

For all GWAS loci used in the standard analysis, we include the associated SNP(s) and a window of all surrounding SNPs into the computation of a single local genetic relatedness matrix (different window sizes were tested, see below). Separately, LD adjustment is performed on each locus individually and then combined into a single GRM to estimate the LD-adjusted heritability (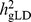, 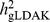). This yields three different models for which the corresponding heritability is then estimated as described above. An alternative strategy of including the GWAS markers as fixed-effects was considered but resulted in under-estimation of the 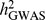 when the fixed and random-effects are highly correlated due to extensive LD between the GWAS variant and surrounding SNPs, and thus was not used.

We also compute local 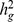 (and corresponding LD-adjusted values) in known GWAS loci from related traits. The procedure is identical to the GWAS analysis but includes only those loci not associated in the focal trait but associated in any related traits. For this autoimmune class, we pool all loci from Celiac disease, Crohn's disease, Graves' disease, Multiple sclerosis, Psoriasis, Rheumatoid arthritis, Systemic lupus erythematosus, Type 1 diabetes, and Ulcerative colitis that are reported in the NCBI catalog. In all cases, total 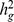, 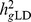, or 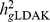 is used where appropriate.

#### Statistical significance of increase in local 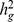


For hypothesis testing, we compute the local expectation as 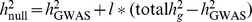 where 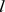 is the physical fraction of the genome corresponding to these loci. We report the relative increase of 
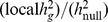
 and compute the statistical significance of this increase by Z-test using the analytical standard error (see above). The same procedure is performed for the LD-adjusted estimates 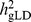 and 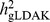 with corresponding genome-wide 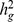 estimates. This approximation based on physical fraction can be biased if the SNP density or LD properties of GWAS loci are substantially different from the rest of the genome. To investigate this bias, we computed local expectation using two alternative measures of 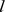: % of SNPs; and % of SNP variance after transforming to the LD-residual. The latter metric is computed as the sum of individual SNP variances after LD-residual regression divided by the corresponding genome-wide sum. This results in regions of high-LD having a reduced contribution compared to physical or SNP size to account for the presence of redundant SNPs. We find these measures to be highly correlated across the nine traits (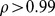 between either metric and 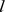 based on physical length), though the % of LD-residual SNP variance is generally higher. However, though the absolute increase in cross-trait analysis did vary across the different metrics (because 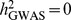 and 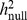 is therefore directly related to 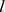) the measures of statistical significance remained consistent and all previously significant estimates remained significant (Table S27).

We do not model the noise on 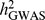 because both 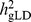 and 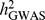 are estimated from the same set of SNPs resulting in these models being partially nested and not independent. We demonstrate this by generating 200 random simulations with a single typed causal variant and regressing 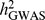 on 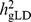 with an intercept, yielding a highly significant effect at 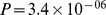 and an 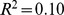. Likewise, we do not model the error around 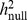 because we are quantifying the observed enrichment of 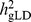 within the same samples that were used to estimate 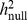. The high concordance of our analytical p-values and those established empirically by sampling random regions (see below) confirm that this assumption is valid.

To ensure that our choice of window size did not significantly impact the results, we performed both the within-trait and related-trait analysis in the WTCCC data while varying window-sizes from 100 Kbp - 2 Mbp (Figure S6,S7). For the within-trait GWAS analysis, the increase in heritability is primarily dependent on the 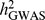 and is therefore stable across all windows. On the other hand, for related-trait analysis the increase in heritability is primarily dependent on the window size, and we observe this strong relationship in the real data. However, we found the significance of increase (computed by z-test) to be stable across the windows tested, and therefore present results from 1 Mbp windows in our main analysis (Figure S6,S7). The LD-residual adjustment is performed in a left-to-right sliding window and could therefore be subtly impacted by SNP ordering. We also re-ran both the trait-specific and related-trait analysis with 1 Mbp window parameters but SNP order reversed, yielding results that were nearly identical (average absolute difference of 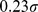) which we do not expect to impact our results.

#### Empirical estimates of significance

Computing significance based on the analytical standard error of each measure of 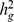 assumes that the analytical standard error is both well-calibrated and normally distributed, and that the genome-wide 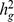 is uniform throughout the genome. We relax these assumptions by using an empirical expectation from randomly sampled regions and comparing the randomly observed enrichment to our observed enrichment at GWAS loci. Specifically, for each trait we randomly draw a number of 1 Mbp regions equal to the number of GWAS loci tested in that trait and compute 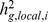 and 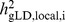 at the union of these loci, performing 1,000 such draws per trait with replacement (or 10,000 for highly significant traits, marked with asterisk). We then compare the 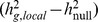 observed at GWAS loci (Table S18) to the sampled 
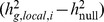
 (conservatively assuming that the sampled 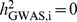) and compute an empirical p-value equal to the number of sampled differences that exceed the observed difference (and likewise for 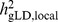). This comparison quantifies how likely the increase we observe in local heritability at GWAS loci is expected to occur by chance at random loci in the genome. Like traditional permutation testing, this procedure does not make assumption of normality, but also captures the true underlying disease architecture of each trait. For the cross-trait analysis, we follow the same procedure but require the random draws to not overlap with known GWAS loci for that trait, as in the real cross-trait analysis.

We find that the resulting empirical p-values are highly consistent with our analytical approximations (Table S17, Figure S5), with the latter appearing over-conservative for the standard 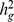 case. All instances that were previously significant (within-trait: CD, UC, and MS; related-trait: UC, CD, RA, T1D) remain significant in the empirical analysis, with T1D also reaching 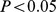 in the within-trait analysis. MS, which was highly significant by z-test in both instances, did not result in any random samples that were more enriched (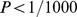). Overall, the strong consistency between these assumption-free estimates lend validity to our analytical approximations of significance. Based on this concordance and the technical restriction on empirical p-values to a minimum of 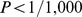 or 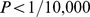, we report the analytical p-values in our main results.

#### Correcting for genic enrichment at GWAS loci

In the related trait analysis, our primary negative control is the lack of significant enrichment among the unrelated traits. However, one potential concern is that a latent correlation between known GWAS loci and gene-coding regions could falsely inflate the relative increase if gene-coding regions systematically contribute more to phenotype. Indeed, recent work partitioning heritability has shown that SNPs near exons contribute significantly more to genome wide 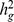 than others [62]. Following the analysis of [62], we define “genic” as any marker within 10 kbp of known exons. Averaged across all data sets, genic SNPs account for 50% of all SNPs and 60% of SNPs in known autoimmune loci, a strong enrichment (Table S21). We computed the fraction of genome-wide 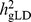 from these genic regions using a joint variance-component model where SNPs from the two region types are modeled in two corresponding components and heritability is estimated jointly. On average, we found that 64% of the total joint estimate comes from the coding component (Table S21). To account for this enrichment, we modify our computation from 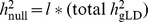 to a weighted average of the two region types:
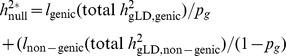
where 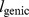 is the fraction of genome covered and genic; 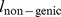 is the fraction of genome covered and non-genic; 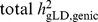 is the variance explained by jointly modeled genic regions; 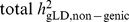 is the variance explained by jointly modeled non-genic regions; and 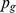 is the fraction of the whole-genome thats genic. Using this new local expectation, we re-calculated the relative increase of local 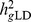 (Table S21). Although the relative increase is slightly lower after this adjustment, all of the traits that were previously significant remain significant and the overall trend persists.

#### PCA-based matching

Multiple Sclerosis has previously been shown to have a high degree of population structure correlated with the trait, which could uniquely bias the MS heritability analysis. To guard against this, we also compute all of our local heritability statistics in cases and controls that have been matched by their top principal components. The matching is performed by reweighing each of the 20 eigenvectors based on each of their 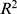 to the phenotype and then computing a Euclidean distance between cases and controls based on the reweighed eigenvectors. For each control, we greedily select the nearest case sample in this space and retain the pair of samples for analysis, iterating until no pair of samples is available. This procedure corresponds to a (greedy) pair match, demonstrated by [63] to effectively control for population structure. After matching and excluding outliers, a total of 8,149 samples were retained with no apparent differences in underlying structure among the main principal components (Figure S8). Local heritability analysis on these matched samples did not yield substantially different results from the full dataset (Table S28), though it did substantially increase the 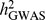. For consistency, we included the original 20 principal components as fixed-effects in the analysis to account for any lingering population structure that was not captured by the matching.

### Simulated quantitative trait loci

#### Genome-wide

For simulations involving genome-wide estimates of heritability and the impact of LD, we used the WTCCC1:CAD cohort to simulate phenotypes and infer components of heritability with the previously described methods. We sampled 5,000 of the genotyped SNPs to be causal variants such that a fraction 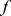 of the markers is low-frequency (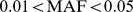), varying 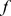 between 0 and 1 in increments of 0.1. We applied allelic effect-sizes drawn from a distribution with mean zero and variance 1/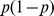 where 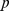 is the variant allele frequency. We generated quantitative phenotypes using the polygenic model with normally-distributed residual variance added to achieve an 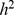 of 0.80. In all simulations the causal variants were always present in the GRM.

#### Local

We estimated the expected effectiveness of the variance-components strategy to identify additional local heritability beyond the GWAS SNP by simulating phenotypes over real genotypes from the analyzed platforms. We emulate the disease architecture identified by Lango-Allen et. al [4], where 180 loci explained approximately 10% of the variance in height. Over multiple trials, we randomly sample 180 1 Mbp loci from the SNP data centered on 1, 2, 3, 5 or 10 casual variants in each locus. The variants are all selected either from minor allele frequency below 5% (low-frequency) or above 10% (common) to create possible disease architectures. For each disease class and locus set combination, we generated quantitative phenotypes using the polygenic model with normalized SNP effect sizes drawn from the standard normal and normally-distributed residual variance added to achieve an 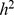 of 0.1 (such that each SNP explains equal phenotypic variance in expectation). The causal variants were then hidden from subsequent analysis and local 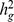 (or LD-adjusted 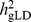 and 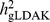) estimated.

For each locus, we specify the “GWAS SNP” to be the single best tag of the true causal variants. In instances where multiple causal variants are present at a locus, this tag is the one SNP with highest unbiased effect-size. Formally, given that each locus 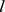 contains set 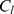 of (at most 10) causal variants and 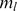 typed GWAS SNPs, we compute the effect of the GWAS SNP as:
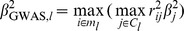
This value represents an idealized scenario where the GWAS SNP explains the most phenotypic variance at the locus with no sampling noise. The heritability 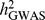 is then calculated as a sum over all 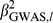.

For the local 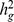, 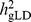, and 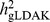 we construct adjusted and unadjusted GRMs over the entire set of typed SNPs and estimated their total contribution to heritability using the standard REML approach described previously. All analyses were performed over 50 independent trials in WTCCC1-CAD (Table S5) and WTCCC2-UC (Table 2) data with randomized causal SNP effect sizes.

#### Local in ImmunoChip

This procedure was modified for simulations in ImmunoChip, requiring the “GWAS SNP” to be selected only from variants in the WTCCC1-RA post-QC data. This reflects the same constraint applied in the real-data analysis, which focused only on associated loci that overlap between the WTCCC RA samples and the ImmunoChip. The result is a marked decrease of 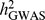 compared to either of the WTCCC simulations, particularly at low-frequency variants (Table S23). Intuitively, this is due to the fact that a causal SNP drawn from the WTCCC array is more likely to be tagged by another WTCCC SNP than a causal SNP drawn from all existing SNPs. The comprehensive assay of variants on the ImmunoChip thus yields simulated causal SNPs that are not as well tagged by WTCCC SNPs as simulations on the WTCCC data itself. For a disease model where causal variants are randomly drawn from all low-frequency SNPs, the ImmunoChip simulations are therefore more representative of real-life GWAS tagging effectiveness. Likewise, we observe an 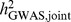 computed over all SNPs that is approximately equal to that observed in WTCCC data for a single causal variant but higher on average over multiple causal variants due to a greater pool of potential SNP tags.

#### Diverse simulations with imputed variants

To investigate more thoroughly the impact of causal allele frequency on components of local heritability we performed a set of simulations using the 1,000 Genomes imputed SNPs in a realistic small-scale analysis of 28 loci with total 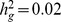 (corresponding to the mean we observed in the WTCCC data). As before, we simulated disease architectures with 1, 2, 3, 5, and 10 causal variants per locus but allowed the causal SNPs to be sampled from the genotyped and 1,000 Genomes imputed markers. We then inferred the local components using either genotyped SNPs only or genotyped and imputed SNPs together. Finally, we performed these simulations in two large runs with the underlying allelic effect-sizes drawn from either the standard normal or a distribution with mean zero and variance 1/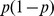 where 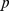 is the variant allele frequency (such that each causal SNP explains equal phenotypic variance in expectation, as in the main Results section). These two architectures are expected under a model of no selection and very strong selection, respectively, providing us with estimates from the two most extreme scenarios. In each simulation, we compared the performance of the following five methods at maximally recovering the local heritability (when causals are hidden) or quantifying it without bias (when causals are observed): 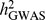, 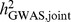, 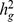, 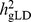, 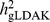.

Tables S8,S29 detail the results for observed causals with the frequency-normalized architecture, again demonstrating the significant deflation of the standard 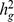 estimates when causal variants are rare (inferring only 0.67 of the true local heritability on average) and inflation when causal variants are common (inferring 1.10 of the true local heritability on average). The two LD adjustment strategies both account for these biases, exhibiting no upwards inflation and no statistically significant differences between the two. For the LD adjusted methods, including imputed SNPs in the analysis increases the observed heritability but not the gain relative to 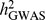 yielding an overall decrease in power to detect additional heritability. These trends were also consistent in simulations of normally distributed effect sizes (Tables S9,S30) with only 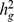 performing better due to the lessened impact of low-frequency variants. When variants were hidden (Tables S6,S10,S7,S11) we again see that two LD adjustment schemes to have no statistically significant differences and lose power when incorporating imputed variants. Both disease architectures yield comparable results.

Like the genome-wide simulations, we conclude that LD adjustment is a necessary step to getting well-controlled estimates, though we no longer observe a significant difference between the two adjustment methods (LD residual and LDAK). Across all simulations, we see that these methods can detect significantly more heritability when multiple causal variants exist at the locus and yield only slightly higher estimates when there is a single causal SNP.

### Web resources

Open-source software implementing the LD residual adjustment we have described is implemented in EIGENSOFT 5.0 at http://www.hsph.harvard.edu/alkes-price/software. HAPI-UR software is available at https://code.google.com/p/hapi-ur/ GCTA software is available at http://www.complextraitgenomics.com/software/gcta/

## Supporting Information

Figure S1Impact of allele frequency on 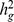 estimate (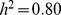 **).** Five strategies for computing 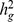 are compared under a disease architecture with 10,000 causal variants increasingly selected from low-frequency SNPs (x-axis). Top panel shows results from phenotypes simulated on 270,000 real WTCCC1-CAD SNPs, bottom panel shows results from phenotypes simulated on 3,900,000 typed and 1,000 Genomes imputed SNPs. Default (IBS) estimate can be slightly inflated or highly deflated depending on disease architecture. Error bars represent observed standard error from 50 random trials.(PDF)

Figure S2Impact of allele frequency on variance of 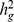 estimate. Four strategies for computing 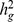 are compared under a disease architecture with 10,000 causal variants increasingly selected from low-frequency SNPs (x-axis). Colored bars represent the mean analytically expected standard error of the SNP-heritability (see Methods) over 50 simulations. White bars represent the observed standard deviation of the estimate over the same simulations.(PDF)

Figure S3

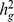
 estimate with no LD (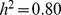). Inference of 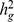 from SNP's randomly permuted to remove LD but maintain allele frequency spectrum. No bias is observed under any tested causal variant frequency distribution.(PDF)

Figure S4 Heritability of liability genome-wide SNPs for seven complex traits. Components of heritability for typed markers (blue) over nine traits and imputed markers (green) over seven WTCCC1 traits shown. Light bars correspond to estimates from the standard variance-component and dark bars correspond to estimate from LD-adjusted variance-component. Two control sub-groups (NBS and 58C) tested against each other as negative control; diseases tested are Bipolar Disorder (BD), Coronary Artery Disease (CAD), Crohn's Disease (CD), Hypertension (HT), Rheumatoid Arthritis (RA), Type 1 Diabetes (T1D), Type 2 Diabetes (T2D), Multiple Sclerosis (MS), Ulcerative Colitis (UC). All traits exhibit an increase after LD adjustment, indicative of a genetic architecture that is shifted towards low-frequency causal variants. Hypertension, which has a family-based estimate of liability-scale heritability close to 1.0, has been hypothesized to be poor fit to the liability-scale transformation [64], and is presented here for completeness.
(PDF)

Figure S5PP-plot for empirical analysis of heritability enrichment. Analytical p-values from estimated 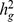 standard error are plotted against empirical p-values estimated from 1,000 randomly sampled regions (10,000 random samplings for phenotypes with asterisk). Top and bottom panels show within and cross-trait analysis; right and left panels show results with and without LD-adjustment. Each p-value position is labeled with the corresponding trait. Dashed red lines indicate significance at 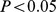 and solid red lines indicate significance after accounting for nine traits. Analysis where no random sample was observed as more enriched are shown at 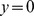. MS was highly significant, with no stronger than random samples observed under any of the four tests, and it is excluded from the plot.(PDF)

Figure S6Increase and Z-score of increase in local heritability measures at known GWAS loci. Components of heritability were inferred around known GWAS loci with a range of locus sizes (100 Kbp - 2 Mbp) and increase compared to 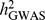 and local expectation is shown. Absolute increase, dependent primarily by 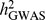 is mostly unaffected by locus size. MS and UC exhibit significant increases at all locus sizes, CD at ≥500 Kbp.(PDF)

Figure S7 Increase and Z-score of increase in local heritability measures at known autoimmune loci. Components of heritability were inferred around known autoimmune disease loci with a range of locus sizes (100 Kbp - 2 Mbp) and increase compared to local expectation is shown. Absolute increase is highly dependent on locus size, however, statistical significance remains largely consistent across all lengths. Autoimmune traits (CD, MS, RA, T1D, and UC) all show statistically significant increases across most locus sizes; non-autoimmune traits (CAD, HT, T2D) show no statistically significant increases under any locus sizes. HLA was excluded from all analyses of autoimmune traits.
(PDF)

Figure S8 PCA of MS samples before and after sample matching. Principal components in MS cohort with highest correlation to phenotype are shown before and after matching samples based on PC coordinates.
(PDF)

Table S1 Datasets analyzed.
(PDF)

Table S2Impact of causal variant allele frequency on fraction of total heritability inferred by five strategies. Each row reports results of five heritability inference strategies from disease architectures where the fraction of causal variants sampled low-frequency (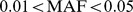) as specified by the left-most column. Other columns report the fraction of total heritability inferred (averaged over 50 trials with standard error in parenthesis), and p-value for difference from 100% by z-test. Bold-face highlights values that are significantly different from 100% by z-test after accounting for 11 tested frequency bins. LD-shrink, LD-residual, and LDAK attempt to account for similar phenomena and their performance is expected to be correlated. Raw estimates are also represented graphically in Figure S1.(PDF)

Table S3Bias in 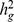 estimates. Summary of the observed bias in simulation for three estimates of 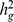. Top row shows results where causal variants are randomly sampled from the genotyped SNPs and bottom row shows corresponding results for non-random sampling of causal variants (from low frequency or high-frequency SNPs). Where significant bias is observed, the range of bias as a fraction of the true 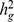 is shown in parenthesis.(PDF)

Table S4RMSE from 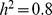 for five LD adjustment schemes.(PDF)

Table S5Fraction of local heritability explained in WTCCC1 simulated phenotypes. Analysis of simulated disease architecture with 180 causal 1 Mbp loci yielding a true 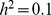. In each locus, 1–10 causal variants were sampled from either low-frequency (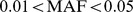) of common (MAF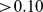) WTCCC1 SNPs. For each of four methods tested, the fraction of local heritability identified by the method is reported over 50 simulations (with standard error in parenthesis). Top two panels correspond to experiments with observed causal variants and bottom two panels to experiments with causal variants hidden. In A and B only (causals are typed), bold-faced 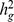 and 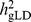 represents significant difference from 100% by z-score at 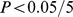 (accounting for 5 architectures tested). The ratio of 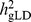 to 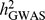 is reported in the bottom row of each panel (with bold-face indicating significance by t-test at 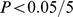).(PDF)

Table S6Fraction of local heritability recovered in simulation (frequency-normalized allelic effect sizes, genotyped SNPs tested). Using 1,000 Genomes imputed variants in the WTCC1:CAD cohort, 28 1 Mbp loci were randomly sampled with every locus centered over a fixed set of causal SNPs (between 1 and 10). Causal variants were sampled from low-frequency (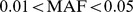, top panel) or common (MAF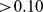, bottom panel) and corresponding allelic effect-sizes were drawn from a normal distribution with mean zero and variance 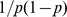 such that each causal SNP explains equal phenotypic variance in expectation. Causal variants were combined as an additive polygenic trait with normally distributed environmental noise set to yield total heritability of 0.02 (number of loci and total heritability chosen as the average over all tested traits in real data). Reported 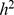 values correspond to the fraction of total heritability recovered by each corresponding method after all causal and imputed variants were hidden, averaged over 100 trails with standard error in parenthesis. 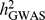 computed from single best tag in the region (see Methods for other models). Gain columns report the ratio of corresponding 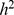 to 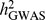, with bold-face indicating significant differences by t-test (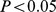). P(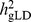 vs. 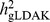) column reports P-value for difference between 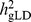 and 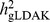 results by Welch's t-test.(PDF)

Table S7Fraction of local heritability recovered in simulation (normal allelic effects, genotyped SNPs tested). Trait simulated and tested as in Table S8 but allelic effect-sizes drawn from a standard normal, such that each causal SNP explains phenotypic variance in proportion to it's allele frequency. Reported 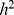 values correspond to the fraction of total heritability (0.02) recovered by each corresponding method, averaged over 50 trails with standard error in parenthesis. Gain columns report the ratio of corresponding 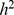 to 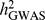, with bold-face indicating significant differences by t-test (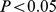). P(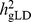 vs. 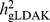) column reports P-value for difference between 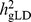 and 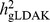 results by Welch's t-test.(PDF)

Table S8Fraction of local heritability observed in simulation (frequency-normalized allelic effect sizes, genotyped SNPs tested). Trait simulated and tested as in Table S6 without hiding causal variants. Reported 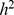 values correspond to the fraction of total heritability (0.02) observed by each corresponding method, averaged over 50 trails with standard error in parenthesis. Gain columns report the ratio of corresponding 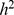 to 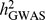, with bold-face indicating significant differences by t-test (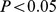). P(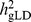 vs. 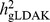) column reports P-value for difference between 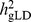 and 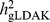 results by Welch's t-test.(PDF)

Table S9Fraction of local heritability observed in simulation (normal allelic effect sizes, genotyped SNPs tested). Trait simulated and tested as in Table S9 without hiding causal variants. Reported 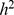 values correspond to the fraction of total heritability (0.02) observed by each corresponding method, averaged over 50 trails with standard error in parenthesis. Gain columns report the ratio of corresponding 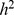 to 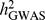, with bold-face indicating significant differences by t-test (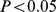). P(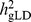 vs. 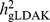) column reports P-value for difference between 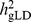 and 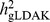 results by Welch's t-test.(PDF)

Table S10Fraction of local heritability recovered in simulation (frequency-normalized allelic effect sizes, imputed SNPs tested). Trait simulated as in Table S6 but heritability recovered from imputed and genotyped SNPs (after hiding causal variants). Reported 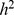 values correspond to the fraction of total heritability (0.02) recovered by each corresponding method, averaged over 100 trails with standard error in parenthesis. Gain columns report the ratio of corresponding 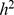 to 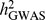, with bold-face indicating significant differences by t-test (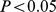). P(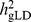 vs. 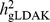) column reports P-value for difference between 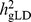 and 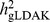 results by Welch's t-test.(PDF)

Table S11Fraction of local heritability recovered in simulation (normal allelic effects, imputed SNPs tested). Trait simulated and tested as in Table S6 but heritability recovered from imputed and genotyped SNPs (after hiding causal variants) and allelic effect-sizes drawn from a standard normal. Reported 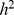 values correspond to the fraction of total heritability (0.02) recovered by each corresponding method, averaged over 50 trails with standard error in parenthesis. Gain columns report the ratio of corresponding 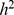 to 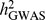, with bold-face indicating significant differences by t-test (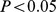). P(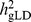 vs. 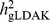) column reports P-value for difference between 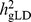 and 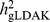 results by Welch's t-test.(PDF)

Table S12Power to detect additional variation in 4,500 samples. Fraction of experiments where specified variance-component estimate (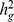 or 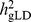) was significantly higher than 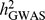 at 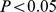 by z-test using analytical standard error on heritability.(PDF)

Table S13Power to detect additional variation in 15,000 samples. Fraction of experiments where specified variance-component estimate (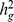 or 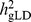) was significantly higher than 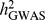 at 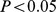 by z-test using analytical standard error on heritability.(PDF)

Table S14Genomewide 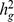 and 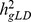 of liability for all case-control traits.(PDF)

Table S15Genomewide observed-scale 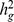 and 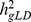 for all case-control traits.(PDF)

Table S16Local heritability around known GWAS loci. Local heritability inferred by LD-adjusted variants components is reported for 1 MBp loci around known GWAS hits for each trait. 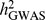 column contains the heritability coming from the top associated SNP at the locus. 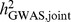 column contains the heritability from all known associated SNPs at the locus and any conditionally significant SNPs (see Methods).(PDF)

Table S17 Analytical and empirical p-values for heritability enrichment. For each locus type and trait, analytical p-values (computed from the Average Information matrix) are compared to empirical p-values (computed from randomly sampled genomic regions). Random sampling was performed over 1,000 trials (10,000 trails for phenotypes marked with asterisk).
(PDF)

Table S18Effect of LD adjustment on heritability around known GWAS loci. Results from three methods for estimating local variance-components are reported, 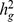 (standard), 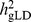 (LD-residual adjusted), and 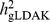 (LDAK adjusted). Gain column reports corresponding 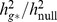, where 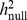 is computed based on the genome-wide 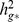 and locus size. P-value computed for each 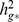 versus corresponding 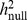 by z-test using analytical standard error.(PDF)

Table S19Heritability of known autoimmune disease loci. Local heritability inferred by LD-adjusted variance-components is reported for known loci associated with autoimmune disease (but not associated for the focal trait). 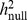 computed from genome-wide 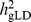 and % of genome. P-value computed for 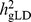 versus 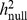 using analytical standard error.(PDF)

Table S20Effect of LD adjustment on heritability of autoimmune disease loci. Results from three methods for estimating local variance-components are reported, 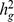 (standard), 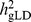 (LD-residual adjusted), and 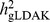 (LDAK adjusted). Gain column reports corresponding 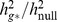, where 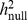 is computed based on the genome-wide 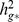 and locus size. P-value computed from z-test using analytical standard error.(PDF)

Table S21Heritability of autoimmune disease loci adjusted for genic enrichment. Enrichment of GWAS loci at genic regions is quantified and adjusted for by recomputing 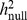 as weighted average of genic and non-genic region size and corresponding total genome-wide 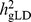. Total 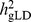 computed from two variance-components for genic and non-genic regions modeled jointly. P-value computed for 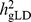 versus adjusted 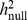 using analytical standard error.(PDF)

Table S22Combined local heritability for autoimmune traits. Estimates of local heritability from known GWAS loci for the respective trait and any known loci for other autoimmune traits are presented separately and together. 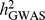 reports the heritability at known GWAS loci for the specified trait. 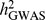 for non-trait autoimmune loci is zero by definition. 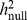 is computed from 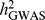 and genome-wide 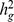. P-values are computed by z-test using the analytical standard error on 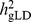.(PDF)

Table S23Fraction of local heritability explained in RACI simulated phenotypes. Analysis of simulated disease architecture with 180 causal 1 Mbp loci yielding a true 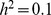. In each locus, 1–10 causal variants were sampled from either low-frequency (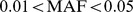) of common (MAF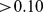) ImmunoChip SNPs and then hidden. For each of four methods tested, the fraction of local heritability identified by the method is reported over 30 simulations (with standard error in parenthesis). 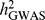 was restricted to SNPs present in WTCCC1 only (consistent with our real analysis). The gain of 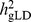 over 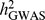 is reported in the bottom row of each panel.(PDF)

Table S24Computation of increased heritability in ImmunoChip data. Four alternative estimates of 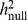 are considered for the ImmunoChip data. Top two panels show enrichment assuming 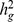 total equals 0.17 as in WTCCC1 or 0.40 as in previously published estimates of 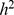. Bottom two panels show the same two assumptions with “flanking” regions estimated from tagging in 1,000 Genomes (see Results). P-value computed for 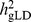 versus corresponding 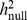 using analytical standard error.(PDF)

Table S25Heritability of previously implicated RA loci in ImmunoChip. Components of local heritability were estimated at two groups of loci suspected in previously published papers. P-value computed for 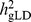 versus corresponding 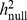 using analytical standard error.(PDF)

Table S26 SNPs analyzed in local variance-components. The number and allele frequency spectrum of SNPs used in local heritability analysis. For known GWAS loci, all genotyped SNPs at the locus as well as any imputed GWAS associated SNPs were included (over the seven WTCCC1 where imputation was performed). For autoimmune loci only genotyped SNPs were included. Effective number of SNPs computed as the sum of SNP variances after performing the LD residual.
(PDF)

Table S27Local heritability enrichment adjusted for SNP density and SNP LD. Three metrics of local expectation are considered: “Physical size”: the fraction of the physical genome taken up by the loci; “SNP size”: the fraction of SNPs within the loci; “LD residual variance”: the fraction of SNP variances in the loci after transforming to LD residuals. For each metric, the local expectation is recomputed and the resulting “Gain” (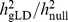) is reported with its corresponding p-value.(PDF)

Table S28 Heritability of MS data with PCA-matched samples. Cases and controls were matched pair-wise based on top 20 principal components (retaining 8,149 samples) and components of local heritability re-estimate.
(PDF)

Table S29Fraction of local heritability observed in simulation (frequency-normalized allelic effect sizes, imputed SNPs tested). Trait simulated and tested as in Table S10 without hiding causal variants. Reported 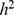 values correspond to the fraction of total heritability (0.02) observed by each corresponding method, averaged over 50 trails with standard error in parenthesis. Gain columns report the ratio of corresponding 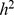 to 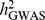, with bold-face indicating significant differences by t-test (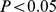). P(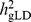 vs. 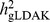) column reports P-value for difference between 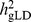 and 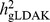 results by Welch's t-test.(PDF)

Table S30Fraction of local heritability observed in simulation (normal allelic effect sizes, imputed SNPs tested). Trait simulated and tested as in Table S11 without hiding causal variants. Reported 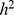 values correspond to the fraction of total heritability (0.02) observed by each corresponding method, averaged over 50 trails with standard error in parenthesis. Gain columns report the ratio of corresponding 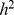 to 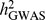, with bold-face indicating significant differences by t-test (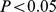). P(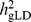 vs. 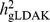) column reports P-value for difference between 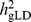 and 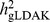 results by Welch's t-test.(PDF)
