# Supplementary figures and images for: Quantifying Missing Heritability at Known GWAS Loci
Source: PLoS Genet. 2013 Dec 26;9(12):e1003993. doi: 10.1371/journal.pgen.1003993 (PMC3873246; doi:10.1371/journal.pgen.1003993)

Figure S1. Impact of allele frequency on  $h_g^2$  estimate ( $h^2 = 0.80$ ).

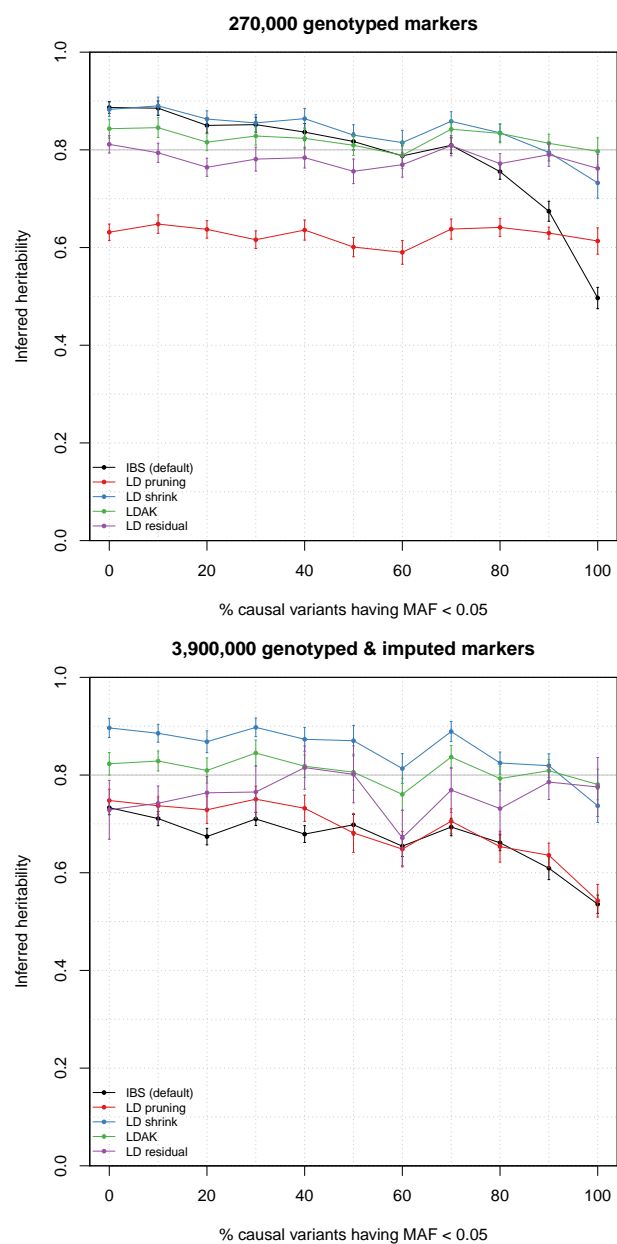

Supplement: Figure S1 — Impact of allele frequency on estimate ( ). Five strategies for computing are compared under a disease architecture with 10,000 causal variants increasingly selected from low-frequency SNPs (x-axis). Top panel shows results from phenotypes simulated on 270,000 real WTCCC1-CAD SNPs, bottom panel shows results from phenotypes simulated on 3,900,000 typed and 1,000 Genomes imputed SNPs. Default (IBS) estimate can be slightly inflated or highly deflated depending on disease architecture. Error bars represent observed standard error from 50 random trials. (PDF) [file pgen.1003993.s001.pdf]

Figure S2. Impact of allele frequency on variance of  $h_g^2$  estimate.

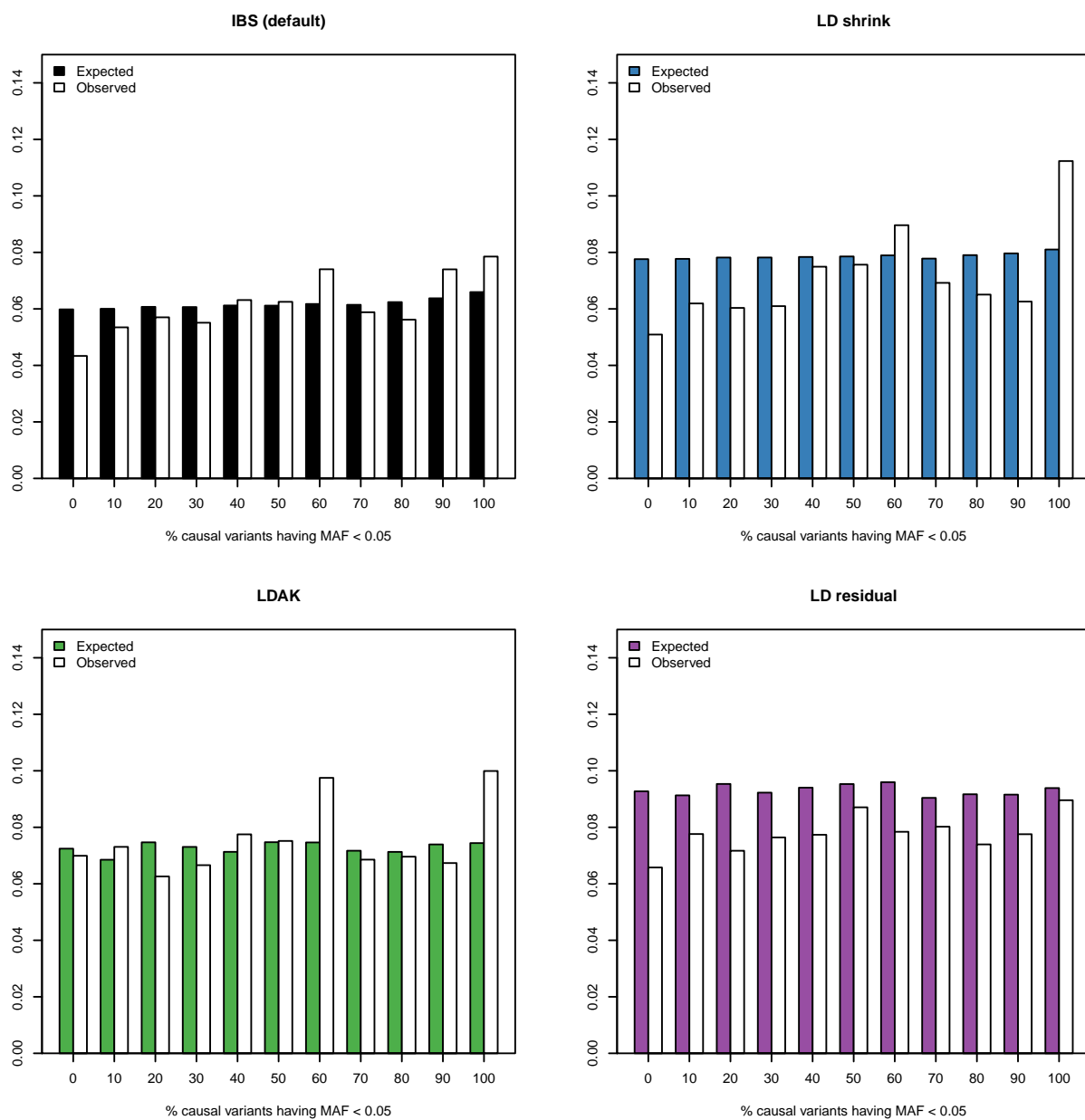

Supplement: Figure S2 — Impact of allele frequency on variance of estimate. Four strategies for computing are compared under a disease architecture with 10,000 causal variants increasingly selected from low-frequency SNPs (x-axis). Colored bars represent the mean analytically expected standard error of the SNP-heritability (see Methods) over 50 simulations. White bars represent the observed standard deviation of the estimate over the same simulations. (PDF) [file pgen.1003993.s002.pdf]

**Figure S3.**  $h_g^2$  estimate with no LD ( $h^2 = 0.80$ ).

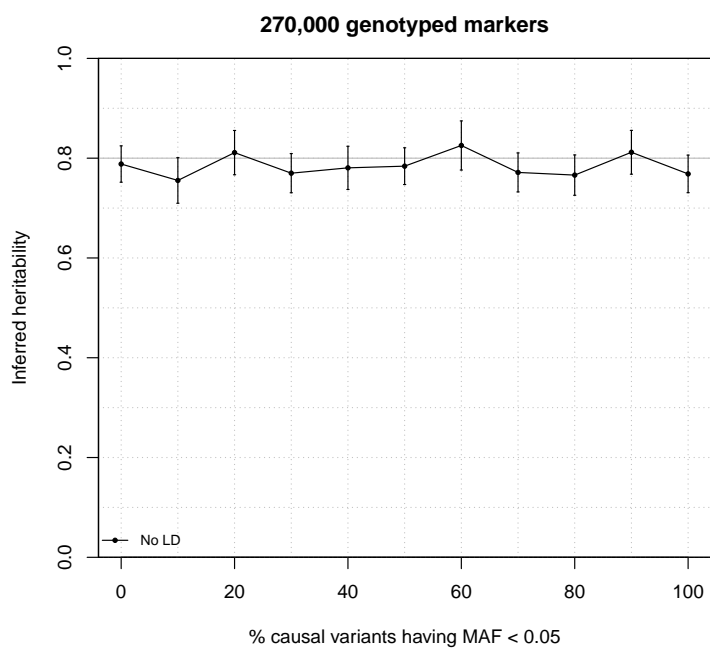

Supplement: Figure S3 — estimate with no LD (). Inference of from SNP's randomly permuted to remove LD but maintain allele frequency spectrum. No bias is observed under any tested causal variant frequency distribution. (PDF) [file pgen.1003993.s003.pdf]

Figure S4. Heritability of liability genome-wide SNPs for seven complex traits.

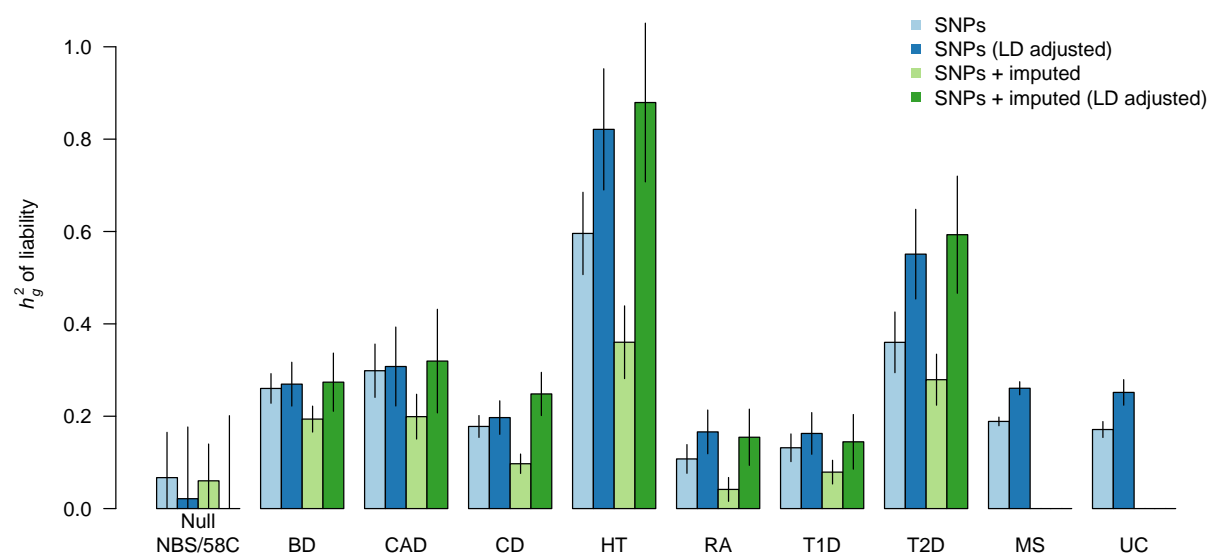

Supplement: Figure S4 — Heritability of liability genome-wide SNPs for seven complex traits. Components of heritability for typed markers (blue) over nine traits and imputed markers (green) over seven WTCCC1 traits shown. Light bars correspond to estimates from the standard variance-component and dark bars correspond to estimate from LD-adjusted variance-component. Two control sub-groups (NBS and 58C) tested against each other as negative control; diseases tested are Bipolar Disorder (BD), Coronary Artery Disease (CAD), Crohn's Disease (CD), Hypertension (HT), Rheumatoid Arthritis (RA), Type 1 Diabetes (T1D), Type 2 Diabetes (T2D), Multiple Sclerosis (MS), Ulcerative Colitis (UC). All traits exhibit an increase after LD adjustment, indicative of a genetic architecture that is shifted towards low-frequency causal variants. Hypertension, which has a family-based estimate of liability-scale heritability close to 1.0, has been hypothesized to be poor fit to the liability-scale transformation [64], and is presented here for completeness. (PDF) [file pgen.1003993.s004.pdf]

Figure S5. PP-plot for empirical analysis of heritability enrichment.

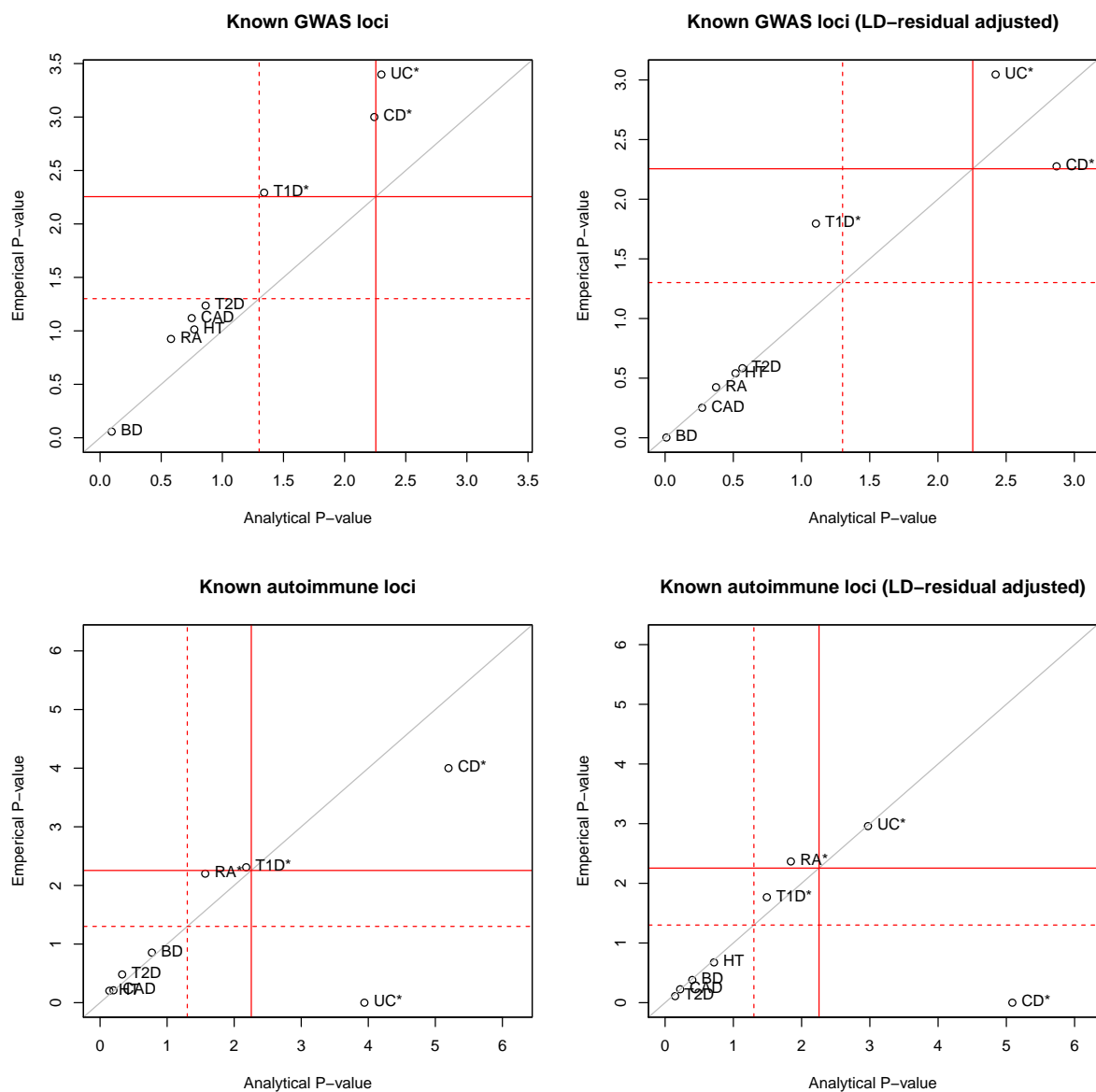

Supplement: Figure S5 — PP-plot for empirical analysis of heritability enrichment. Analytical p-values from estimated standard error are plotted against empirical p-values estimated from 1,000 randomly sampled regions (10,000 random samplings for phenotypes with asterisk). Top and bottom panels show within and cross-trait analysis; right and left panels show results with and without LD-adjustment. Each p-value position is labeled with the corresponding trait. Dashed red lines indicate significance at and solid red lines indicate significance after accounting for nine traits. Analysis where no random sample was observed as more enriched are shown at . MS was highly significant, with no stronger than random samples observed under any of the four tests, and it is excluded from the plot. (PDF) [file pgen.1003993.s005.pdf]

Figure S6. Increase and Z-score of increase in local heritability measures at known GWAS loci.

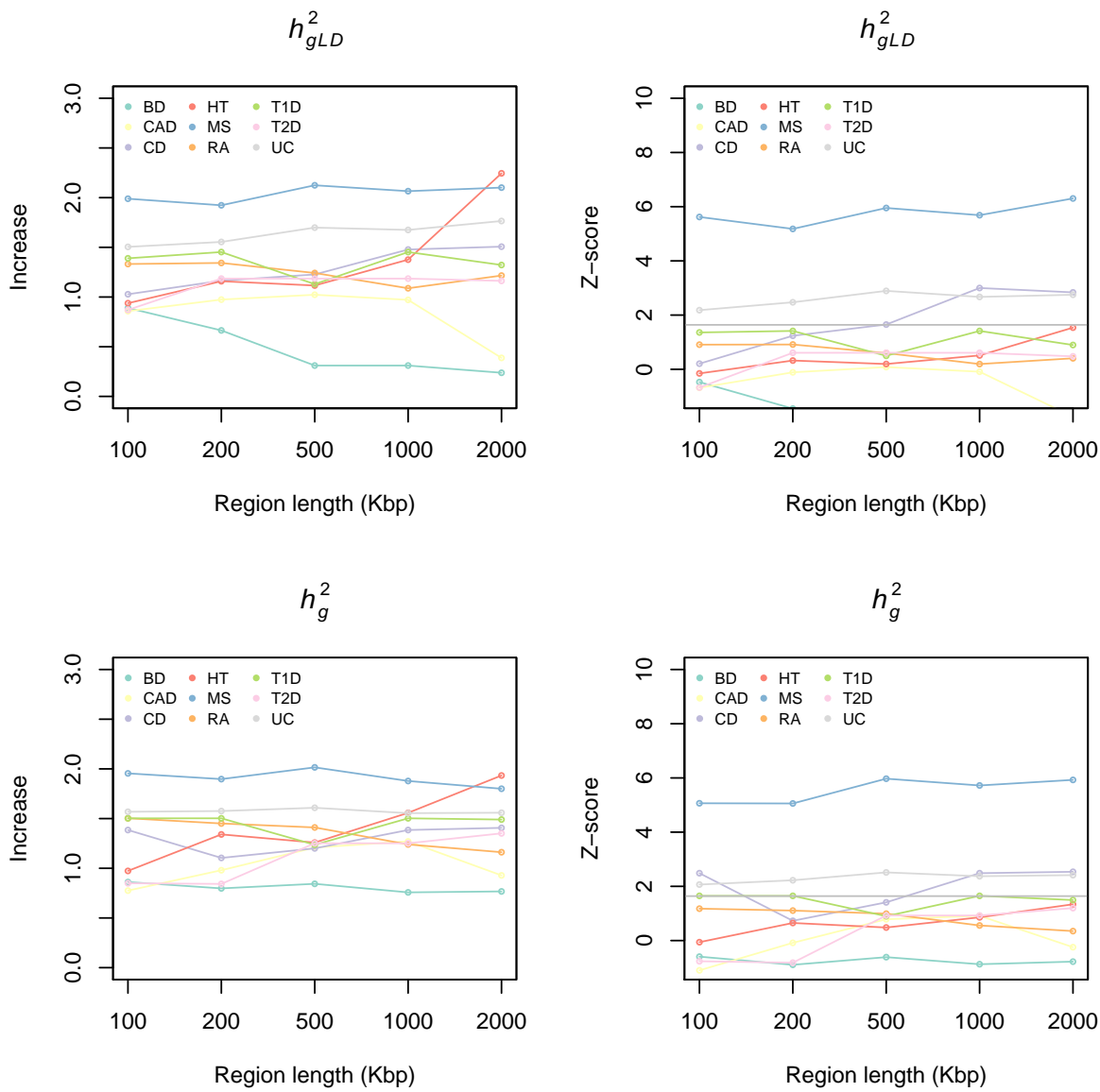

Supplement: Figure S6 — Increase and Z-score of increase in local heritability measures at known GWAS loci. Components of heritability were inferred around known GWAS loci with a range of locus sizes (100 Kbp - 2 Mbp) and increase compared to and local expectation is shown. Absolute increase, dependent primarily by is mostly unaffected by locus size. MS and UC exhibit significant increases at all locus sizes, CD at ≥500 Kbp. (PDF) [file pgen.1003993.s006.pdf]

Figure S8. PCA of MS samples before and after sample matching.

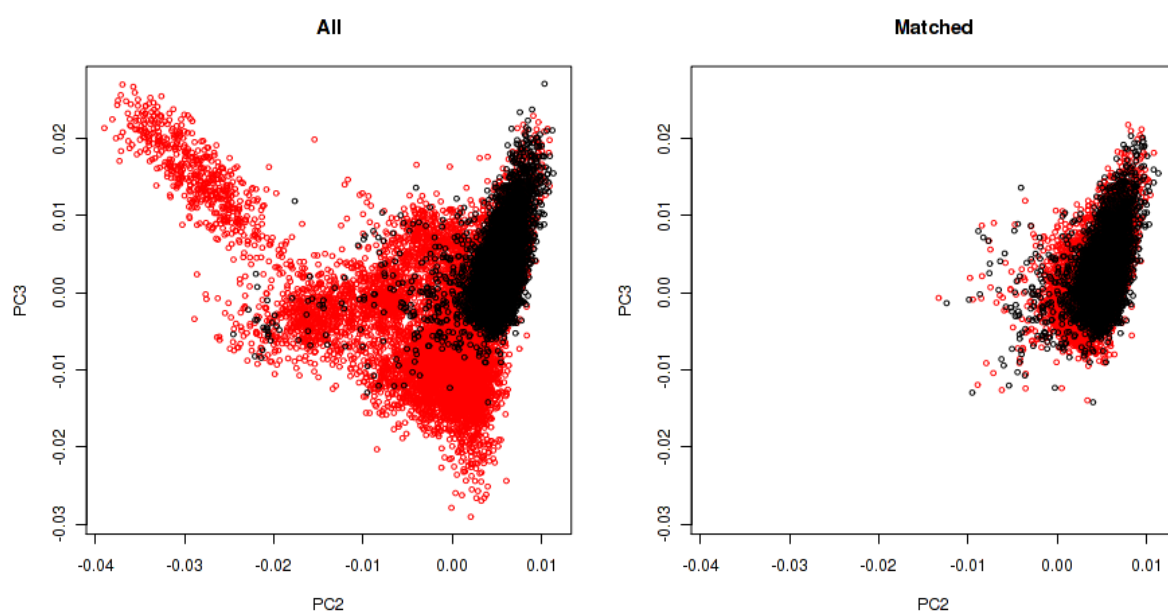

Supplement: Figure S8 — PCA of MS samples before and after sample matching. Principal components in MS cohort with highest correlation to phenotype are shown before and after matching samples based on PC coordinates. (PDF) [file pgen.1003993.s008.pdf]
