# Supplementary material for: Quantifying Missing Heritability at Known GWAS Loci
Source: PLoS Genet. 2013 Dec 26;9(12):e1003993. doi: 10.1371/journal.pgen.1003993 (PMC3873246; doi:10.1371/journal.pgen.1003993)

Figure S7. Increase and Z-score of increase in local heritability measures at known autoimmune loci.

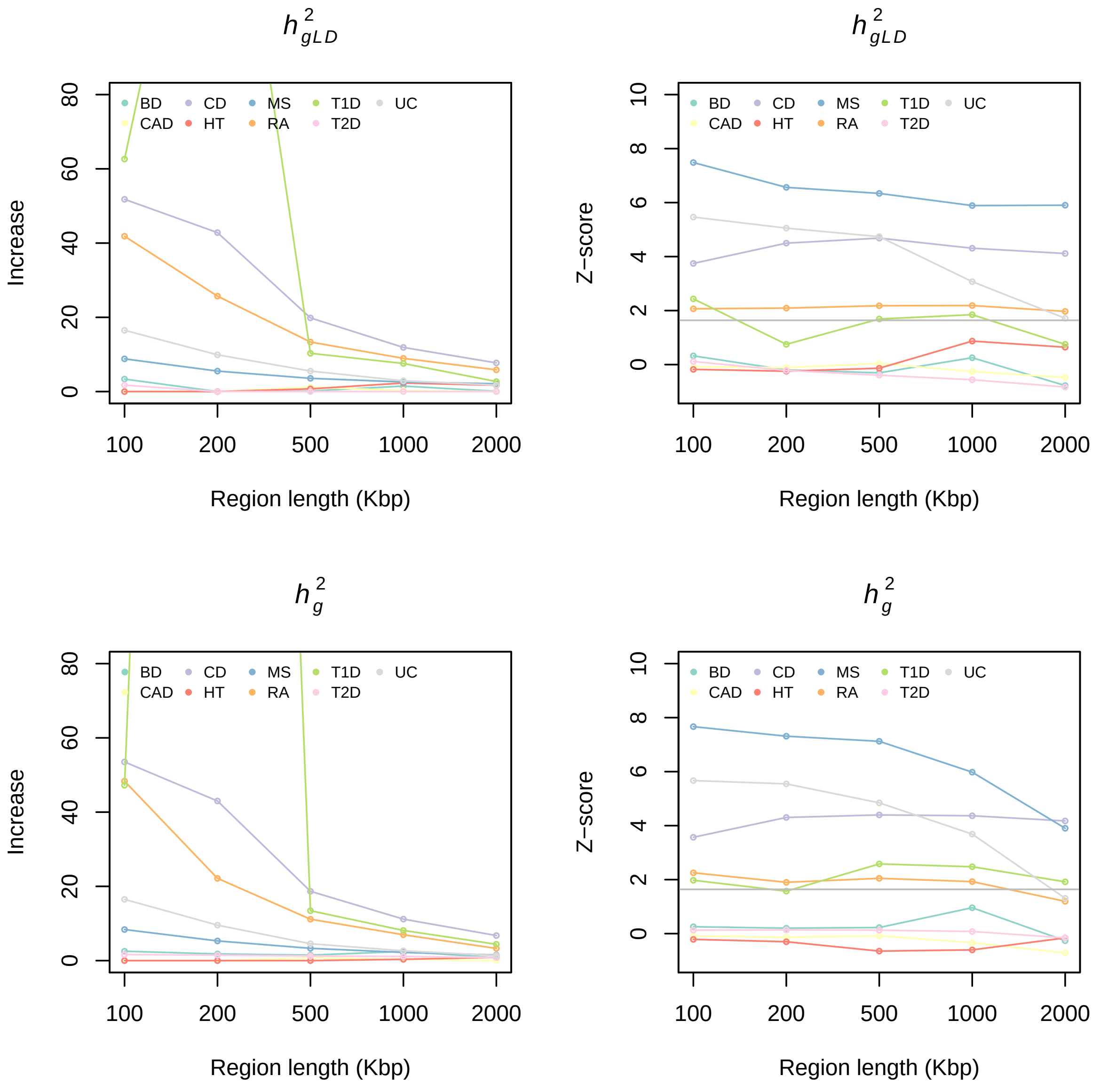

Supplement: Figure S7 — Increase and Z-score of increase in local heritability measures at known autoimmune loci. Components of heritability were inferred around known autoimmune disease loci with a range of locus sizes (100 Kbp - 2 Mbp) and increase compared to local expectation is shown. Absolute increase is highly dependent on locus size, however, statistical significance remains largely consistent across all lengths. Autoimmune traits (CD, MS, RA, T1D, and UC) all show statistically significant increases across most locus sizes; non-autoimmune traits (CAD, HT, T2D) show no statistically significant increases under any locus sizes. HLA was excluded from all analyses of autoimmune traits. (PDF) [file pgen.1003993.s007.pdf]
