# Supplementary material for: Quantifying Missing Heritability at Known GWAS Loci
Source: PLoS Genet. 2013 Dec 26;9(12):e1003993. doi: 10.1371/journal.pgen.1003993 (PMC3873246; doi:10.1371/journal.pgen.1003993)
Supplement: Table S1 — Datasets analyzed. (PDF) [file pgen.1003993.s009.pdf]

Table S1. Datasets analyzed.

Cohort	Prevalence	Samples	SNPs	QC Samples (% Cases)	QC SNPs	Imputed SNPs
BD: Bipolar Disorder	0.005	4806	459446	4216 (36%)	143064	2169252
CAD: Coronary Artery Disease	0.060	4864	459446	4414 (39%)	139576	2174048
CD: Crohns Disease	0.001	4686	459446	4204 (36%)	146962	2175065
HT: Hypertension	0.260	4890	459446	4399 (39%)	139551	2171672
RA: Rheumatoid Arthritis	0.005	4798	459446	4328 (38%)	143742	2168515
T1D: Type 1 Diabetes	0.005	4901	459446	4414 (39%)	139214	2163251
T2D: Type 2 Diabetes	0.080	4862	459446	4312 (38%)	142037	2171935
UC: Ulcerative Colitis	0.001	8349	719635	7923 (31%)	447945	-
MS: Multiple Sclerosis	0.001	16247	547196	14526 (64%)	396509	-
