# Supplementary material for: Quantifying Missing Heritability at Known GWAS Loci
Source: PLoS Genet. 2013 Dec 26;9(12):e1003993. doi: 10.1371/journal.pgen.1003993 (PMC3873246; doi:10.1371/journal.pgen.1003993)
Supplement: Table S2 — Impact of causal variant allele frequency on fraction of total heritability inferred by five strategies. Each row reports results of five heritability inference strategies from disease architectures where the fraction of causal variants sampled low-frequency () as specified by the left-most column. Other columns report the fraction of total heritability inferred (averaged over 50 trials with standard error in parenthesis), and p-value for difference from 100% by z-test. Bold-face highlights values that are significantly different from 100% by z-test after accounting for 11 tested frequency bins. LD-shrink, LD-residual, and LDAK attempt to account for similar phenomena and their performance is expected to be correlated. Raw estimates are also represented graphically in Figure S1. (PDF) [file pgen.1003993.s010.pdf]

Table S2. Impact of causal variant allele frequency on fraction of total heritability inferred by five strategies.

Genotyped SNPs:

% low MAF	Standard		LD pruning		LD shrink		LDAK		LD-residual	
	h_g^2 (se)	P-Value	h_g^2 (se)	P-Value	h_g^2 (se)	P-Value	h_{gLDAK}^2 (se)	P-Value	h_{gLD}^2 (se)	P-Value
0%	**110%** (0.8%)	$< 1.00 \times 10^{-16}$	**78%** (1.1%)	$< 1.00 \times 10^{-16}$	**110%** (0.9%)	$< 1.00 \times 10^{-16}$	**105%** (1.2%)	6.00×10^{-06}	101% (1.1%)	1.00×10^{-01}
10%	**110%** (0.9%)	$< 1.00 \times 10^{-16}$	**81%** (1.2%)	$< 1.00 \times 10^{-16}$	**111%** (1.1%)	$< 1.00 \times 10^{-16}$	**105%** (1.3%)	5.40×10^{-06}	99% (1.3%)	2.70×10^{-01}
20%	**106%** (1.0%)	2.80×10^{-10}	**79%** (1.1%)	$< 1.00 \times 10^{-16}$	**107%** (1.1%)	1.30×10^{-13}	101% (1.1%)	3.80×10^{-02}	**95%** (1.2%)	7.90×10^{-05}
30%	**106%** (1.0%)	1.80×10^{-11}	**76%** (1.1%)	$< 1.00 \times 10^{-16}$	**106%** (1.1%)	1.80×10^{-10}	**103%** (1.2%)	1.30×10^{-03}	97% (1.6%)	6.40×10^{-02}
40%	**104%** (1.1%)	2.30×10^{-05}	**79%** (1.3%)	$< 1.00 \times 10^{-16}$	**107%** (1.3%)	8.40×10^{-10}	102% (1.4%)	1.60×10^{-02}	98% (1.3%)	6.90×10^{-02}
50%	102% (1.1%)	2.60×10^{-02}	**75%** (1.3%)	$< 1.00 \times 10^{-16}$	**103%** (1.4%)	2.60×10^{-03}	101% (1.3%)	1.90×10^{-01}	**94%** (1.6%)	3.00×10^{-04}
60%	98% (1.3%)	1.20×10^{-01}	**73%** (1.6%)	$< 1.00 \times 10^{-16}$	101% (1.6%)	1.20×10^{-01}	98% (1.7%)	2.10×10^{-01}	96% (1.6%)	9.90×10^{-03}
70%	101% (1.0%)	1.30×10^{-01}	**79%** (1.3%)	$< 1.00 \times 10^{-16}$	**107%** (1.2%)	2.50×10^{-09}	**105%** (1.2%)	6.50×10^{-06}	101% (1.3%)	2.10×10^{-01}
80%	**94%** (1.0%)	9.70×10^{-09}	**80%** (1.2%)	$< 1.00 \times 10^{-16}$	**104%** (1.2%)	7.50×10^{-05}	**104%** (1.2%)	3.00×10^{-04}	**96%** (1.3%)	3.40×10^{-03}
90%	**84%** (1.3%)	$< 1.00 \times 10^{-16}$	**78%** (0.8%)	$< 1.00 \times 10^{-16}$	99% (1.1%)	2.80×10^{-01}	101% (1.2%)	8.00×10^{-02}	98% (1.5%)	2.10×10^{-01}
100%	**62%** (1.4%)	$< 1.00 \times 10^{-16}$	**76%** (1.7%)	$< 1.00 \times 10^{-16}$	**91%** (2.0%)	1.00×10^{-05}	99% (1.8%)	4.10×10^{-01}	95% (1.8%)	4.90×10^{-03}

Imputed SNPs:

% low MAF	Standard		LD pruning		LD shrink		LDAK		LD-residual	
	h_g^2 (se)	P-Value	h_g^2 (se)	P-Value	h_g^2 (se)	P-Value	h_{gLDAK}^2 (se)	P-Value	h_{gLD}^2 (se)	P-Value
0%	**91%** (0.9%)	$< 1.00 \times 10^{-16}$	**91%** (0.9%)	$< 1.00 \times 10^{-16}$	**112%** (1.3%)	$< 1.00 \times 10^{-16}$	102% (1.5%)	2.50×10^{-02}	91% (3.8%)	1.00×10^{-02}
10%	**88%** (0.9%)	$< 1.00 \times 10^{-16}$	**88%** (0.9%)	$< 1.00 \times 10^{-16}$	**110%** (1.2%)	$< 1.00 \times 10^{-16}$	**103%** (1.3%)	2.80×10^{-03}	**92%** (2.3%)	7.00×10^{-04}
20%	**84%** (1.1%)	$< 1.00 \times 10^{-16}$	**84%** (1.1%)	$< 1.00 \times 10^{-16}$	**108%** (1.4%)	8.60×10^{-10}	101% (1.6%)	2.40×10^{-01}	95% (2.1%)	1.70×10^{-02}
30%	**88%** (0.9%)	$< 1.00 \times 10^{-16}$	**88%** (0.9%)	$< 1.00 \times 10^{-16}$	**112%** (1.2%)	$< 1.00 \times 10^{-16}$	**105%** (1.7%)	4.40×10^{-04}	95% (3.4%)	1.00×10^{-01}
40%	**84%** (1.1%)	$< 1.00 \times 10^{-16}$	**84%** (1.1%)	$< 1.00 \times 10^{-16}$	**109%** (1.5%)	1.70×10^{-09}	102% (1.5%)	6.30×10^{-02}	101% (2.8%)	2.50×10^{-01}
50%	**87%** (1.3%)	$< 1.00 \times 10^{-16}$	**87%** (1.3%)	$< 1.00 \times 10^{-16}$	**108%** (2.0%)	4.70×10^{-06}	100% (2.4%)	3.80×10^{-01}	100% (3.7%)	4.80×10^{-01}
60%	**81%** (1.4%)	$< 1.00 \times 10^{-16}$	**81%** (1.4%)	$< 1.00 \times 10^{-16}$	101% (1.9%)	2.00×10^{-01}	95% (2.1%)	9.70×10^{-03}	**83%** (3.6%)	4.70×10^{-06}
70%	**86%** (1.1%)	$< 1.00 \times 10^{-16}$	**86%** (1.1%)	$< 1.00 \times 10^{-16}$	**111%** (1.3%)	$< 1.00 \times 10^{-16}$	**104%** (1.5%)	1.00×10^{-03}	96% (2.9%)	9.40×10^{-02}
80%	**82%** (1.0%)	$< 1.00 \times 10^{-16}$	**82%** (1.0%)	$< 1.00 \times 10^{-16}$	103% (1.4%)	1.50×10^{-02}	99% (1.6%)	2.90×10^{-01}	**91%** (3.2%)	3.80×10^{-03}
90%	**76%** (1.5%)	$< 1.00 \times 10^{-16}$	**76%** (1.5%)	$< 1.00 \times 10^{-16}$	102% (1.5%)	6.10×10^{-02}	101% (1.5%)	2.20×10^{-01}	98% (2.3%)	2.20×10^{-01}
100%	**66%** (1.2%)	$< 1.00 \times 10^{-16}$	**66%** (1.2%)	$< 1.00 \times 10^{-16}$	**92%** (2.2%)	1.40×10^{-04}	97% (2.0%)	1.10×10^{-01}	96% (3.9%)	2.10×10^{-01}
