# Supplementary material for: Quantifying Missing Heritability at Known GWAS Loci
Source: PLoS Genet. 2013 Dec 26;9(12):e1003993. doi: 10.1371/journal.pgen.1003993 (PMC3873246; doi:10.1371/journal.pgen.1003993)
Supplement: Table S3 — Bias in estimates. Summary of the observed bias in simulation for three estimates of . Top row shows results where causal variants are randomly sampled from the genotyped SNPs and bottom row shows corresponding results for non-random sampling of causal variants (from low frequency or high-frequency SNPs). Where significant bias is observed, the range of bias as a fraction of the true is shown in parenthesis. (PDF) [file pgen.1003993.s011.pdf]

Table S3. Bias in h_g^2 estimates.

Causal variant sampling	Standard estimate (h_g^2)	LD-residual estimate (h^2_{gLD})	LDAK estimate (h^2_{gLDAK})
Random	Approximately unbiased	Downwards bias (95% of h_g^2)	Approximately unbiased
Non-random	Biased (62% - 110% of h_g^2)	Downwards bias (94% of h_g^2)	Upwards bias (105% of h_g^2)
