# Supplementary material for: Quantifying Missing Heritability at Known GWAS Loci
Source: PLoS Genet. 2013 Dec 26;9(12):e1003993. doi: 10.1371/journal.pgen.1003993 (PMC3873246; doi:10.1371/journal.pgen.1003993)
Supplement: Table S4 — RMSE from for five LD adjustment schemes. (PDF) [file pgen.1003993.s012.pdf]

Table S4. RMSE from $h^2 = 0.8$ for five LD adjustment schemes.

Adjustment	Genotyped	Imputed
LD pruning	0.175	0.128
Standard (IBS)	0.109	0.141
LD shrink	0.058	0.070
LDAK	0.028	0.026
LD residual	0.026	0.056
