# Supplementary material for: Quantifying Missing Heritability at Known GWAS Loci
Source: PLoS Genet. 2013 Dec 26;9(12):e1003993. doi: 10.1371/journal.pgen.1003993 (PMC3873246; doi:10.1371/journal.pgen.1003993)
Supplement: Table S5 — Fraction of local heritability explained in WTCCC1 simulated phenotypes. Analysis of simulated disease architecture with 180 causal 1 Mbp loci yielding a true . In each locus, 1–10 causal variants were sampled from either low-frequency () of common (MAF) WTCCC1 SNPs. For each of four methods tested, the fraction of local heritability identified by the method is reported over 50 simulations (with standard error in parenthesis). Top two panels correspond to experiments with observed causal variants and bottom two panels to experiments with causal variants hidden. In A and B only (causals are typed), bold-faced and represents significant difference from 100% by z-score at (accounting for 5 architectures tested). The ratio of to is reported in the bottom row of each panel (with bold-face indicating significance by t-test at ). (PDF) [file pgen.1003993.s013.pdf]

**Table S5. Fraction of local heritability explained in WTCCC1 simulated phenotypes.**

| | # Low-frequency typed causals: | | | | |
|---|---|---|---|---|---|
| A: | 1 | 2 | 3 | 5 | 10 |
| $h^2_{\text{GWAS}}$ | 100% | 81% | 70% | 55% | 37% |
| $h^2_{\text{GWAS,joint}}$ | 101% | 86% | 75% | 60% | 46% |
| $h^2_g$ local (se) | **60%** (3%) | **66%** (3%) | **71%** (3%) | **71%** (2%) | **87%** (3%) |
| $h^2_{g\text{LD}}$ local (se) | 102% (4%) | 107% (3%) | 106% (3%) | 103% (3%) | 103% (4%) |
| $h^2_{g\text{LD}}/h^2_{\text{GWAS}}$ | 1.03 | **1.32** | **1.52** | **1.86** | **2.76** |
| | # Common typed causals: | | | | |
| B: | 1 | 2 | 3 | 5 | 10 |
| $h^2_{\text{GWAS}}$ | 100% | 82% | 70% | 56% | 37% |
| $h^2_{\text{GWAS,joint}}$ | 101% | 84% | 71% | 58% | 45% |
| $h^2_g$ local (se) | 106% (3%) | 104% (2%) | 102% (2%) | **109%** (3%) | **110%** (2%) |
| $h^2_{g\text{LD}}$ local (se) | 97% (3%) | 89% (3%) | 92% (3%) | 100% (3%) | 102% (5%) |
| $h^2_{g\text{LD}}/h^2_{\text{GWAS}}$ | 0.98 | 1.09 | **1.32** | **1.79** | **2.70** |
| | # Low-frequency untyped causals: | | | | |
| C: | 1 | 2 | 3 | 5 | 10 |
| $h^2_{\text{GWAS}}$ | 39% | 32% | 29% | 21% | 14% |
| $h^2_{\text{GWAS,joint}}$ | 40% | 35% | 34% | 24% | 20% |
| $h^2_g$ local (se) | 28% (2%) | 29% (2%) | 31% (2%) | 24% (2%) | 23% (3%) |
| $h^2_{g\text{LD}}$ local (se) | 47% (3%) | 49% (3%) | 52% (3%) | 41% (3%) | 37% (4%) |
| $h^2_{g\text{LD}}/h^2_{\text{GWAS}}$ | 1.21 | **1.51** | **1.80** | **1.93** | **2.57** |
| | # Common untyped causals: | | | | |
| D: | 1 | 2 | 3 | 5 | 10 |
| $h^2_{\text{GWAS}}$ | 66% | 55% | 46% | 39% | 26% |
| $h^2_{\text{GWAS,joint}}$ | 68% | 58% | 46% | 42% | 32% |
| $h^2_g$ local (se) | 80% (3%) | 80% (2%) | 75% (2%) | 84% (2%) | 80% (2%) |
| $h^2_{g\text{LD}}$ local (se) | 77% (3%) | 69% (3%) | 66% (3%) | 78% (3%) | 77% (4%) |
| $h^2_{g\text{LD}}/h^2_{\text{GWAS}}$ | **1.16** | **1.25** | **1.44** | **1.99** | **2.89** |
