# Supplementary material for: Quantifying Missing Heritability at Known GWAS Loci
Source: PLoS Genet. 2013 Dec 26;9(12):e1003993. doi: 10.1371/journal.pgen.1003993 (PMC3873246; doi:10.1371/journal.pgen.1003993)
Supplement: Table S6 — Fraction of local heritability recovered in simulation (frequency-normalized allelic effect sizes, genotyped SNPs tested). Using 1,000 Genomes imputed variants in the WTCC1:CAD cohort, 28 1 Mbp loci were randomly sampled with every locus centered over a fixed set of causal SNPs (between 1 and 10). Causal variants were sampled from low-frequency (, top panel) or common (MAF, bottom panel) and corresponding allelic effect-sizes were drawn from a normal distribution with mean zero and variance such that each causal SNP explains equal phenotypic variance in expectation. Causal variants were combined as an additive polygenic trait with normally distributed environmental noise set to yield total heritability of 0.02 (number of loci and total heritability chosen as the average over all tested traits in real data). Reported values correspond to the fraction of total heritability recovered by each corresponding method after all causal and imputed variants were hidden, averaged over 100 trails with standard error in parenthesis. computed from single best tag in the region (see Methods for other models). Gain columns report the ratio of corresponding to , with bold-face indicating significant differences by t-test (). P( vs. ) column reports P-value for difference between and results by Welch's t-test. (PDF) [file pgen.1003993.s014.pdf]

Table S6. Fraction of local heritability recovered in simulation (frequency-normalized allelic effect sizes, genotyped SNPs tested).

# casuals	h^2_{GWAS}	$h^2_{\text{GWAS,joint}}$	Gain	h^2_{g}	Gain	h^2_{gLD}	Gain	h^2_{gLDAK}	Gain	P(h^2_{gLD} vs. h^2_{gLDAK})
Low-frequency un-typed causal variants:										
1	0.21	0.23 (0.02)	1.10	0.09 (0.04)	**0.41**	0.24 (0.05)	1.15	0.18 (0.05)	0.87	3.9×10^{-01}
2	0.29	0.29 (0.02)	1.00	0.33 (0.04)	1.13	0.43 (0.06)	**1.47**	0.41 (0.05)	**1.42**	8.7×10^{-01}
3	0.23	0.25 (0.02)	1.10	0.33 (0.04)	**1.43**	0.38 (0.05)	**1.66**	0.39 (0.05)	**1.68**	9.4×10^{-01}
5	0.22	0.25 (0.02)	1.12	0.37 (0.04)	**1.69**	0.44 (0.05)	**1.99**	0.41 (0.04)	**1.87**	6.8×10^{-01}
10	0.16	0.22 (0.01)	**1.35**	0.25 (0.04)	**1.57**	0.33 (0.05)	**2.07**	0.32 (0.05)	**2.01**	8.9×10^{-01}
Average	0.22	0.25 (0.05)	1.12	0.27 (0.05)	1.24	0.36 (0.05)	1.65	0.34 (0.05)	1.56	
Common un-typed causal variants:										
# casuals	h^2_{GWAS}	$h^2_{\text{GWAS,joint}}$	Gain	h^2_{g}	Gain	h^2_{gLD}	Gain	h^2_{gLDAK}	Gain	P(h^2_{gLD} vs. h^2_{gLDAK})
1	0.70	0.73 (0.02)	1.04	0.80 (0.04)	**1.14**	0.68 (0.05)	0.97	0.74 (0.04)	1.06	3.6×10^{-01}
2	0.59	0.62 (0.02)	1.05	0.85 (0.05)	**1.45**	0.72 (0.05)	**1.21**	0.81 (0.05)	**1.37**	2.3×10^{-01}
3	0.52	0.57 (0.02)	**1.10**	0.95 (0.05)	**1.82**	0.82 (0.06)	**1.58**	0.90 (0.05)	**1.73**	3.3×10^{-01}
5	0.43	0.50 (0.02)	**1.16**	0.92 (0.04)	**2.15**	0.75 (0.05)	**1.74**	0.87 (0.04)	**2.03**	4.0×10^{-02}
10	0.28	0.38 (0.02)	**1.37**	0.78 (0.04)	**2.80**	0.63 (0.05)	**2.26**	0.68 (0.05)	**2.42**	5.2×10^{-01}
Average	0.50	0.56 (0.05)	1.12	0.86 (0.05)	1.72	0.72 (0.05)	1.44	0.80 (0.05)	1.60	
