# Supplementary material for: Quantifying Missing Heritability at Known GWAS Loci
Source: PLoS Genet. 2013 Dec 26;9(12):e1003993. doi: 10.1371/journal.pgen.1003993 (PMC3873246; doi:10.1371/journal.pgen.1003993)
Supplement: Table S7 — Fraction of local heritability recovered in simulation (normal allelic effects, genotyped SNPs tested). Trait simulated and tested as in Table S8 but allelic effect-sizes drawn from a standard normal, such that each causal SNP explains phenotypic variance in proportion to it's allele frequency. Reported values correspond to the fraction of total heritability (0.02) recovered by each corresponding method, averaged over 50 trails with standard error in parenthesis. Gain columns report the ratio of corresponding to , with bold-face indicating significant differences by t-test (). P( vs. ) column reports P-value for difference between and results by Welch's t-test. (PDF) [file pgen.1003993.s015.pdf]

Table S7. Fraction of local heritability recovered in simulation (normal allelic effects, genotyped SNPs tested).

Low-frequency un-typed causal variants:										
# casuals	h^2_{GWAS}	$h^2_{\text{GWAS,joint}}$	Gain	h^2_{g}	Gain	h^2_{gLD}	Gain	h^2_{gLDAK}	Gain	P(h^2_{gLD} vs. h^2_{gLDAK})
1	0.23	0.26 (0.03)	1.15	0.26 (0.06)	1.14	0.37 (0.08)	1.62	0.24 (0.05)	1.02	1.7×10^{-01}
2	0.29	0.36 (0.02)	**1.25**	0.30 (0.05)	1.04	0.40 (0.07)	1.39	0.40 (0.05)	**1.38**	9.8×10^{-01}
3	0.26	0.31 (0.02)	1.20	0.31 (0.05)	1.19	0.52 (0.07)	**1.99**	0.33 (0.06)	1.26	4.2×10^{-02}
5	0.22	0.25 (0.02)	1.12	0.26 (0.06)	1.18	0.36 (0.07)	1.65	0.37 (0.06)	**1.68**	9.4×10^{-01}
10	0.16	0.24 (0.02)	**1.50**	0.25 (0.06)	1.56	0.43 (0.07)	**2.66**	0.36 (0.07)	**2.26**	4.9×10^{-01}
Average	0.23	0.28 (0.07)	1.24	0.28 (0.07)	1.20	0.42 (0.07)	1.81	0.34 (0.07)	1.47	
**Common un-typed causal variants:**										
# casuals	h^2_{GWAS}	$h^2_{\text{GWAS,joint}}$	Gain	h^2_{g}	Gain	h^2_{gLD}	Gain	h^2_{gLDAK}	Gain	P(h^2_{gLD} vs. h^2_{gLDAK})
1	0.68	0.71 (0.03)	1.05	0.80 (0.07)	1.18	0.69 (0.08)	1.01	0.82 (0.07)	1.20	2.1×10^{-01}
2	0.58	0.59 (0.03)	1.02	0.75 (0.06)	**1.29**	0.69 (0.08)	1.18	0.81 (0.07)	**1.39**	2.8×10^{-01}
3	0.51	0.59 (0.03)	**1.15**	0.80 (0.05)	**1.57**	0.76 (0.07)	**1.48**	0.83 (0.06)	**1.64**	3.8×10^{-01}
5	0.44	0.49 (0.03)	1.11	0.75 (0.07)	**1.72**	0.68 (0.09)	**1.54**	0.80 (0.06)	**1.82**	2.5×10^{-01}
10	0.25	0.38 (0.02)	**1.52**	0.76 (0.05)	**3.06**	0.68 (0.07)	**2.72**	0.82 (0.07)	**3.29**	1.4×10^{-01}
Average	0.49	0.55 (0.07)	1.13	0.77 (0.07)	1.58	0.70 (0.07)	1.42	0.82 (0.07)	1.67	
