# Supplementary material for: Quantifying Missing Heritability at Known GWAS Loci
Source: PLoS Genet. 2013 Dec 26;9(12):e1003993. doi: 10.1371/journal.pgen.1003993 (PMC3873246; doi:10.1371/journal.pgen.1003993)
Supplement: Table S9 — Fraction of local heritability observed in simulation (normal allelic effect sizes, genotyped SNPs tested). Trait simulated and tested as in Table S9 without hiding causal variants. Reported values correspond to the fraction of total heritability (0.02) observed by each corresponding method, averaged over 50 trails with standard error in parenthesis. Gain columns report the ratio of corresponding to , with bold-face indicating significant differences by t-test (). P( vs. ) column reports P-value for difference between and results by Welch's t-test. (PDF) [file pgen.1003993.s017.pdf]

**Table S9. Fraction of local heritability observed in simulation (normal allelic effect sizes, genotyped SNPs tested).**

Low-frequency un-typed causal variants:

| # casuals | $h^2_{\text{GWAS}}$ | $h^2_{\text{GWAS,joint}}$ | Gain | $h^2_{\text{g}}$ | Gain | $h^2_{\text{gLD}}$ | Gain | $h^2_{\text{gLDAK}}$ | Gain | P($h^2_{\text{gLD}}$ vs. $h^2_{\text{gLDAK}}$) |
|---|---|---|---|---|---|---|---|---|---|---|
| 1 | 1.01 | 1.04 (0.03) | 1.03 | 0.62 (0.07) | **0.62** | 1.13 (0.08) | 1.12 | 0.93 (0.06) | 0.93 | $5.8 \times 10^{-02}$ |
| 2 | 0.82 | 0.86 (0.03) | 1.05 | 0.63 (0.06) | **0.77** | 1.01 (0.08) | **1.23** | 1.00 (0.06) | **1.22** | $9.3 \times 10^{-01}$ |
| 3 | 0.71 | 0.69 (0.03) | 0.98 | 0.65 (0.06) | 0.92 | 1.04 (0.07) | **1.47** | 1.00 (0.07) | **1.41** | $6.9 \times 10^{-01}$ |
| 5 | 0.56 | 0.61 (0.03) | 1.09 | 0.72 (0.07) | **1.28** | 1.00 (0.08) | **1.79** | 0.96 (0.06) | **1.71** | $6.8 \times 10^{-01}$ |
| 10 | 0.38 | 0.49 (0.03) | **1.29** | 0.87 (0.07) | **2.29** | 1.03 (0.08) | **2.72** | 0.94 (0.07) | **2.47** | $3.9 \times 10^{-01}$ |
| Average | 0.70 | 0.74 (0.07) | 1.05 | 0.70 (0.07) | 1.00 | 1.04 (0.07) | 1.49 | 0.97 (0.07) | 1.38 | |

Common un-typed causal variants:

| # casuals | $h^2_{\text{GWAS}}$ | $h^2_{\text{GWAS,joint}}$ | Gain | $h^2_{\text{g}}$ | Gain | $h^2_{\text{gLD}}$ | Gain | $h^2_{\text{gLDAK}}$ | Gain | P($h^2_{\text{gLD}}$ vs. $h^2_{\text{gLDAK}}$) |
|---|---|---|---|---|---|---|---|---|---|---|
| 1 | 1.00 | 1.00 (0.03) | 1.00 | 1.04 (0.07) | 1.04 | 0.88 (0.07) | 0.88 | 1.02 (0.08) | 1.02 | $2.0 \times 10^{-01}$ |
| 2 | 0.82 | 0.81 (0.03) | 0.99 | 0.98 (0.06) | **1.20** | 0.82 (0.08) | 1.00 | 0.96 (0.07) | 1.17 | $2.2 \times 10^{-01}$ |
| 3 | 0.71 | 0.76 (0.03) | 1.07 | 1.08 (0.06) | **1.52** | 0.95 (0.07) | **1.34** | 0.98 (0.06) | **1.38** | $7.9 \times 10^{-01}$ |
| 5 | 0.56 | 0.64 (0.03) | **1.15** | 1.03 (0.07) | **1.84** | 0.83 (0.09) | **1.48** | 0.97 (0.06) | **1.73** | $2.1 \times 10^{-01}$ |
| 10 | 0.37 | 0.45 (0.02) | **1.23** | 1.04 (0.05) | **2.82** | 0.90 (0.07) | **2.43** | 1.00 (0.07) | **2.71** | $2.9 \times 10^{-01}$ |
| Average | 0.69 | 0.73 (0.07) | 1.06 | 1.04 (0.07) | 1.50 | 0.88 (0.07) | 1.27 | 0.98 (0.07) | 1.43 | |
