# Supplementary material for: Quantifying Missing Heritability at Known GWAS Loci
Source: PLoS Genet. 2013 Dec 26;9(12):e1003993. doi: 10.1371/journal.pgen.1003993 (PMC3873246; doi:10.1371/journal.pgen.1003993)
Supplement: Table S10 — Fraction of local heritability recovered in simulation (frequency-normalized allelic effect sizes, imputed SNPs tested). Trait simulated as in Table S6 but heritability recovered from imputed and genotyped SNPs (after hiding causal variants). Reported values correspond to the fraction of total heritability (0.02) recovered by each corresponding method, averaged over 100 trails with standard error in parenthesis. Gain columns report the ratio of corresponding to , with bold-face indicating significant differences by t-test (). P( vs. ) column reports P-value for difference between and results by Welch's t-test. (PDF) [file pgen.1003993.s018.pdf]

Table S10. Fraction of local heritability recovered in simulation (frequency-normalized allelic effect sizes, imputed SNPs tested).

# casuals	h^2_{GWAS}	$h^2_{\mathrm{GWAS,joint}}$	Gain	h^2_{g}	Gain	h^2_{gLD}	Gain	h^2_{gLDAK}	Gain	P(h^2_{gLD} vs. h^2_{gLDAK})
Low-frequency un-typed causal variants:										
1	0.69	0.69 (0.02)	0.99	0.46 (0.04)	**0.66**	0.74 (0.08)	1.07	0.64 (0.07)	0.93	3.9×10^{-01}
2	0.67	0.66 (0.02)	0.99	0.57 (0.04)	**0.85**	0.70 (0.08)	1.05	0.82 (0.07)	**1.23**	2.4×10^{-01}
3	0.52	0.55 (0.02)	1.07	0.62 (0.05)	**1.20**	0.79 (0.09)	**1.52**	0.80 (0.06)	**1.54**	9.4×10^{-01}
5	0.44	0.48 (0.02)	**1.08**	0.61 (0.04)	**1.39**	0.80 (0.07)	**1.82**	0.82 (0.06)	**1.87**	8.3×10^{-01}
10	0.32	0.37 (0.02)	**1.16**	0.58 (0.04)	**1.81**	0.56 (0.07)	**1.74**	0.71 (0.06)	**2.21**	1.2×10^{-01}
Average	0.53	0.55 (0.06)	1.04	0.57 (0.06)	1.07	0.72 (0.06)	1.35	0.76 (0.06)	1.43	
Common un-typed causal variants:										
# casuals	h^2_{GWAS}	$h^2_{\mathrm{GWAS,joint}}$	Gain	h^2_{g}	Gain	h^2_{gLD}	Gain	h^2_{gLDAK}	Gain	P(h^2_{gLD} vs. h^2_{gLDAK})
1	0.83	0.86 (0.02)	1.04	0.69 (0.04)	**0.84**	0.84 (0.08)	1.02	0.84 (0.06)	1.01	9.8×10^{-01}
2	0.74	0.78 (0.02)	1.05	0.84 (0.05)	1.13	0.75 (0.08)	1.02	0.96 (0.07)	**1.30**	5.9×10^{-02}
3	0.62	0.66 (0.02)	1.07	0.81 (0.04)	**1.31**	1.01 (0.08)	**1.63**	1.06 (0.07)	**1.71**	6.4×10^{-01}
5	0.51	0.54 (0.02)	1.06	0.87 (0.04)	**1.71**	0.80 (0.08)	**1.56**	0.99 (0.05)	**1.94**	3.4×10^{-02}
10	0.34	0.42 (0.02)	**1.25**	0.78 (0.04)	**2.29**	0.76 (0.08)	**2.23**	0.83 (0.06)	**2.44**	4.7×10^{-01}
Average	0.61	0.65 (0.06)	1.07	0.80 (0.06)	1.31	0.83 (0.06)	1.36	0.94 (0.06)	1.54	
