# Supplementary material for: Quantifying Missing Heritability at Known GWAS Loci
Source: PLoS Genet. 2013 Dec 26;9(12):e1003993. doi: 10.1371/journal.pgen.1003993 (PMC3873246; doi:10.1371/journal.pgen.1003993)
Supplement: Table S11 — Fraction of local heritability recovered in simulation (normal allelic effects, imputed SNPs tested). Trait simulated and tested as in Table S6 but heritability recovered from imputed and genotyped SNPs (after hiding causal variants) and allelic effect-sizes drawn from a standard normal. Reported values correspond to the fraction of total heritability (0.02) recovered by each corresponding method, averaged over 50 trails with standard error in parenthesis. Gain columns report the ratio of corresponding to , with bold-face indicating significant differences by t-test (). P( vs. ) column reports P-value for difference between and results by Welch's t-test. (PDF) [file pgen.1003993.s019.pdf]

Table S11. Fraction of local heritability recovered in simulation (normal allelic effects, imputed SNPs tested).

Low-frequency un-typed causal variants:										
# casuals	h^2_{GWAS}	$h^2_{\mathrm{GWAS,joint}}$	Gain	h^2_{g}	Gain	h^2_{gLD}	Gain	h^2_{gLDAK}	Gain	P(h^2_{gLD} vs. h^2_{gLDAK})
1	0.71	0.76 (0.03)	1.08	0.63 (0.06)	0.88	0.87 (0.11)	1.23	0.65 (0.08)	0.91	1.1×10^{-01}
2	0.67	0.71 (0.03)	1.06	0.56 (0.06)	0.84	0.84 (0.09)	1.25	0.86 (0.08)	**1.28**	8.4×10^{-01}
3	0.53	0.55 (0.03)	1.05	0.55 (0.05)	1.04	0.81 (0.11)	**1.53**	0.69 (0.10)	1.30	4.2×10^{-01}
5	0.45	0.55 (0.03)	**1.22**	0.67 (0.06)	**1.49**	0.59 (0.10)	1.31	0.77 (0.08)	**1.71**	1.6×10^{-01}
10	0.32	0.41 (0.03)	**1.29**	0.68 (0.07)	**2.14**	0.73 (0.10)	**2.28**	0.73 (0.10)	**2.27**	9.8×10^{-01}
Average	0.54	0.60 (0.10)	1.11	0.62 (0.10)	1.14	0.77 (0.10)	1.42	0.74 (0.10)	1.37	
Common un-typed causal variants:										
# casuals	h^2_{GWAS}	$h^2_{\mathrm{GWAS,joint}}$	Gain	h^2_{g}	Gain	h^2_{gLD}	Gain	h^2_{gLDAK}	Gain	P(h^2_{gLD} vs. h^2_{gLDAK})
1	0.84	0.86 (0.03)	1.02	0.68 (0.07)	**0.81**	0.82 (0.11)	0.97	0.92 (0.10)	1.10	4.7×10^{-01}
2	0.73	0.74 (0.03)	1.02	0.69 (0.06)	0.95	0.85 (0.11)	1.17	0.96 (0.10)	**1.31**	4.6×10^{-01}
3	0.63	0.64 (0.03)	1.02	0.62 (0.06)	0.99	0.84 (0.09)	**1.33**	0.92 (0.09)	**1.47**	5.0×10^{-01}
5	0.51	0.58 (0.02)	**1.13**	0.72 (0.06)	**1.41**	0.74 (0.11)	**1.44**	0.94 (0.07)	**1.84**	1.2×10^{-01}
10	0.28	0.40 (0.02)	**1.41**	0.74 (0.05)	**2.65**	0.80 (0.09)	**2.87**	0.94 (0.09)	**3.37**	2.6×10^{-01}
Average	0.60	0.64 (0.09)	1.07	0.69 (0.09)	1.15	0.81 (0.09)	1.35	0.94 (0.09)	1.56	
