# Supplementary material for: Quantifying Missing Heritability at Known GWAS Loci
Source: PLoS Genet. 2013 Dec 26;9(12):e1003993. doi: 10.1371/journal.pgen.1003993 (PMC3873246; doi:10.1371/journal.pgen.1003993)
Supplement: Table S12 — Power to detect additional variation in 4,500 samples. Fraction of experiments where specified variance-component estimate ( or ) was significantly higher than at by z-test using analytical standard error on heritability. (PDF) [file pgen.1003993.s020.pdf]

Table S12. Power to detect additional variation in 4,500 samples.

Frequency	$h^2_{g\mathrm{LD}}$	h^2_g
1 causal variant		
Low-frequency	0.04	0.02
Common	0.08	0.10
2 causal variant		
Low-frequency	0.04	0.00
Common	0.02	0.22
3 causal variant		
Low-frequency	0.12	0.08
Common	0.10	0.34
5 causal variant		
Low-frequency	0.12	0.00
Common	0.26	0.62
10 causal variant		
Low-frequency	0.16	0.16
Common	0.56	0.74
