# Supplementary material for: Quantifying Missing Heritability at Known GWAS Loci
Source: PLoS Genet. 2013 Dec 26;9(12):e1003993. doi: 10.1371/journal.pgen.1003993 (PMC3873246; doi:10.1371/journal.pgen.1003993)
Supplement: Table S13 — Power to detect additional variation in 15,000 samples. Fraction of experiments where specified variance-component estimate ( or ) was significantly higher than at by z-test using analytical standard error on heritability. (PDF) [file pgen.1003993.s021.pdf]

Table S13. Power to detect additional variation in 15,000 samples.

Frequency	$h^2_{g\mathrm{LD}}$	h^2_g
1 causal variant		
uncommon	0.250	0.000
common	0.100	0.400
2 causal variants		
uncommon	0.400	0.050
common	0.300	0.700
3 causal variants		
uncommon	0.900	0.100
common	0.850	1.000
