# Supplementary material for: Quantifying Missing Heritability at Known GWAS Loci
Source: PLoS Genet. 2013 Dec 26;9(12):e1003993. doi: 10.1371/journal.pgen.1003993 (PMC3873246; doi:10.1371/journal.pgen.1003993)
Supplement: Table S14 — Genomewide and of liability for all case-control traits. (PDF) [file pgen.1003993.s022.pdf]

Table S14. Genomewide h_g^2 and h_{gLD}^2 of liability for all case-control traits.

	Genotyped		Genotyped & imputed	
Phenotype	h_g^2 total (se)	$h_{g\mathrm{LD}}^2$ total (se)	h_g^2 total (se)	$h_{g\mathrm{LD}}^2$ total (se)
BD	0.26 (0.032)	0.27 (0.047)	0.19 (0.028)	0.27 (0.063)
CAD	0.30 (0.058)	0.31 (0.085)	0.20 (0.048)	0.32 (0.112)
CD	0.18 (0.024)	0.20 (0.036)	0.10 (0.021)	0.25 (0.047)
HT	0.60 (0.089)	0.82 (0.131)	0.36 (0.079)	0.88 (0.172)
RA	0.11 (0.031)	0.17 (0.047)	0.04 (0.026)	0.15 (0.061)
T1D	0.13 (0.030)	0.16 (0.045)	0.08 (0.026)	0.14 (0.059)
T2D	0.36 (0.066)	0.55 (0.097)	0.28 (0.055)	0.59 (0.127)
UC	0.17 (0.017)	0.25 (0.028)	-	-
MS	0.19 (0.009)	0.26 (0.014)	-	-
