# Supplementary material for: Quantifying Missing Heritability at Known GWAS Loci
Source: PLoS Genet. 2013 Dec 26;9(12):e1003993. doi: 10.1371/journal.pgen.1003993 (PMC3873246; doi:10.1371/journal.pgen.1003993)
Supplement: Table S15 — Genomewide observed-scale and for all case-control traits. (PDF) [file pgen.1003993.s023.pdf]

Table S15. Genomewide observed-scale h_g^2 and h_{gLD}^2 for all case-control traits.

	Genotyped		Genotyped & imputed	
Phenotype	h_g^2 total (se)	$h_{g\mathrm{LD}}^2$ total (se)	h_g^2 total (se)	$h_{g\mathrm{LD}}^2$ total (se)
NBS/58C Controls	0.07 (0.098)	0.02 (0.155)	0.06 (0.080)	0.00 (0.201)
BD	0.51 (0.063)	0.53 (0.093)	0.38 (0.055)	0.54 (0.124)
CAD	0.32 (0.062)	0.33 (0.091)	0.21 (0.052)	0.34 (0.120)
CD	0.47 (0.063)	0.52 (0.096)	0.26 (0.055)	0.66 (0.123)
HT	0.40 (0.060)	0.56 (0.089)	0.24 (0.053)	0.60 (0.117)
RA	0.21 (0.062)	0.33 (0.095)	0.08 (0.052)	0.31 (0.122)
T1D	0.27 (0.061)	0.33 (0.091)	0.16 (0.052)	0.29 (0.119)
T2D	0.35 (0.063)	0.53 (0.093)	0.27 (0.053)	0.57 (0.122)
UC	0.42 (0.042)	0.62 (0.068)	-	-
MS	0.49 (0.024)	0.68 (0.037)	-	-
