# Supplementary material for: Quantifying Missing Heritability at Known GWAS Loci
Source: PLoS Genet. 2013 Dec 26;9(12):e1003993. doi: 10.1371/journal.pgen.1003993 (PMC3873246; doi:10.1371/journal.pgen.1003993)
Supplement: Table S16 — Local heritability around known GWAS loci. Local heritability inferred by LD-adjusted variants components is reported for 1 MBp loci around known GWAS hits for each trait. column contains the heritability coming from the top associated SNP at the locus. column contains the heritability from all known associated SNPs at the locus and any conditionally significant SNPs (see Methods). (PDF) [file pgen.1003993.s024.pdf]

## Table S16. Local heritability around known GWAS loci.

| | | GWAS | | | Joint markers | | | $h^2_{\text{gLD}}$ Local | | | |
|---|---|---|---|---|---|---|---|---|---|---|---|
| Phenotype | Total $h^2_{g\text{LD}}$ | Loci | % genome | $h^2_{\text{GWAS}}$ | SNPs[a] | $h^2_{\text{GWAS,joint}}$ | Increase | Expected[b] | Observed (se) | $h^2_{\text{gLD}}/h^2_{\text{null}}$ [b] | P-value[c] |
| BD | 0.27 | 21 | 0.9% | 0.014 | 27 | 0.015 | 1.08 | 0.016 | 0.005 (0.005) | 0.31 | $9.8 \times 10^{-01}$ |
| CAD | 0.31 | 26 | 1.1% | 0.032 | 33 | 0.032 | 1.00 | 0.035 | 0.034 (0.011) | 0.97 | $5.4 \times 10^{-01}$ |
| CD | 0.20 | 35 | 1.4% | 0.037 | 57 | 0.043 | 1.15 | 0.040 | 0.059 (0.006) | 1.48 | $1.3 \times 10^{-03}$ |
| HT | 0.82 | 12 | 0.5% | 0.012 | 13 | 0.013 | 1.05 | 0.016 | 0.022 (0.012) | 1.38 | $3.0 \times 10^{-01}$ |
| RA | 0.17 | 13 | 0.5% | 0.008 | 17 | 0.013 | 1.59 | 0.009 | 0.010 (0.004) | 1.09 | $4.2 \times 10^{-01}$ |
| T1D | 0.16 | 17 | 0.7% | 0.016 | 24 | 0.020 | 1.23 | 0.018 | 0.025 (0.006) | 1.45 | $7.8 \times 10^{-02}$ |
| T2D | 0.55 | 27 | 1.1% | 0.039 | 33 | 0.039 | 1.00 | 0.045 | 0.053 (0.014) | 1.19 | $2.7 \times 10^{-01}$ |
| UC | 0.25 | 21 | 0.8% | 0.012 | 28 | 0.016 | 1.28 | 0.014 | 0.024 (0.004) | 1.68 | $3.8 \times 10^{-03}$ |
| MS | 0.26 | 77 | 3.1% | 0.012 | 92 | 0.014 | 1.15 | 0.020 | 0.041 (0.004) | 2.07 | $6.5 \times 10^{-09}$ |
| Average: | | | | | | | 1.17 | | | 1.29 | Combined: $3.3 \times 10^{-05}$ |

[a]Number of significant SNPs selected in joint model.
[b]Fold-increase compared to expectation given $h^2_{\text{GWAS}}$ and fraction of genome covered.
[c]Computed by z-test of $h^2_{\text{gLD,local}}$ against $h^2_{\text{null}}$ using analytical standard error.
