# Supplementary material for: Quantifying Missing Heritability at Known GWAS Loci
Source: PLoS Genet. 2013 Dec 26;9(12):e1003993. doi: 10.1371/journal.pgen.1003993 (PMC3873246; doi:10.1371/journal.pgen.1003993)
Supplement: Table S17 — Analytical and empirical p-values for heritability enrichment. For each locus type and trait, analytical p-values (computed from the Average Information matrix) are compared to empirical p-values (computed from randomly sampled genomic regions). Random sampling was performed over 1,000 trials (10,000 trails for phenotypes marked with asterisk). (PDF) [file pgen.1003993.s025.pdf]

Table S17. Analytical and empirical p-values for heritability enrichment.

GWAS Loci:				
	$h^2_{\mathrm{g,local}}$		$h^2_{\mathrm{gLD,local}}$	
Phenotype	Analytical P	Empirical P	Analytical P	Empirical P
BD	8.0×10^{-01}	8.8×10^{-01}	9.8×10^{-01}	9.9×10^{-01}
CAD	1.8×10^{-01}	7.6×10^{-02}	5.4×10^{-01}	5.6×10^{-01}
CD*	5.7×10^{-03}	1.0×10^{-03}	1.3×10^{-03}	5.3×10^{-03}
HT	1.7×10^{-01}	9.7×10^{-02}	3.0×10^{-01}	2.9×10^{-01}
RA	2.6×10^{-01}	1.2×10^{-01}	4.2×10^{-01}	3.8×10^{-01}
T1D*	4.6×10^{-02}	5.1×10^{-03}	7.8×10^{-02}	1.6×10^{-02}
T2D	1.4×10^{-01}	5.8×10^{-02}	2.7×10^{-01}	2.6×10^{-01}
UC*	5.0×10^{-03}	4.0×10^{-04}	3.8×10^{-03}	9.0×10^{-04}
MS	5.5×10^{-11}	$< 1.0 \times 10^{-03}$	6.5×10^{-09}	$< 1.0 \times 10^{-03}$

Autoimmune Loci:				
	$h^2_{\mathrm{g,local}}$		$h^2_{\mathrm{gLD,local}}$	
Phenotype	Analytical P	Empirical P	Analytical P	Empirical P
MS	1.1×10^{-09}	$< 1.0 \times 10^{-03}$	1.9×10^{-09}	$< 1.0 \times 10^{-03}$
UC*	1.1×10^{-04}	$< 1.0 \times 10^{-04}$	1.1×10^{-03}	1.1×10^{-03}
CD*	6.4×10^{-06}	1.0×10^{-04}	8.2×10^{-06}	$< 1.0 \times 10^{-04}$
RA*	2.7×10^{-02}	6.3×10^{-03}	1.4×10^{-02}	4.3×10^{-03}
T1D*	6.6×10^{-03}	4.9×10^{-03}	3.2×10^{-02}	1.7×10^{-02}
BD	1.7×10^{-01}	1.4×10^{-01}	4.0×10^{-01}	4.2×10^{-01}
CAD	6.3×10^{-01}	6.1×10^{-01}	6.0×10^{-01}	6.0×10^{-01}
HT	7.3×10^{-01}	6.3×10^{-01}	1.9×10^{-01}	2.1×10^{-01}
T2D	4.7×10^{-01}	3.3×10^{-01}	7.1×10^{-01}	7.8×10^{-01}
