# Supplementary material for: Quantifying Missing Heritability at Known GWAS Loci
Source: PLoS Genet. 2013 Dec 26;9(12):e1003993. doi: 10.1371/journal.pgen.1003993 (PMC3873246; doi:10.1371/journal.pgen.1003993)
Supplement: Table S18 — Effect of LD adjustment on heritability around known GWAS loci. Results from three methods for estimating local variance-components are reported, (standard), (LD-residual adjusted), and (LDAK adjusted). Gain column reports corresponding , where is computed based on the genome-wide and locus size. P-value computed for each versus corresponding by z-test using analytical standard error. (PDF) [file pgen.1003993.s026.pdf]

Table S18. Effect of LD adjustment on heritability around known GWAS loci.

Phenotype	h^2_g	h^2_{gLD}	h^2_{gLDAK}	h^2_{GWAS}	Unadjusted			LD-residual Adjusted			LDAK Adjusted		
					h^2_g (se)	Gain	P-value	h^2_{gLD} (se)	Gain	P-value	h^2_{gLDAK} (se)	Gain	P-value
BD	0.26	0.27	0.28	0.014	0.012 (0.004)	0.76	8.0×10^{-01}	0.005 (0.005)	0.31	9.8×10^{-01}	0.009 (0.005)	0.59	9.1×10^{-01}
CAD	0.30	0.31	0.30	0.032	0.045 (0.010)	1.27	1.8×10^{-01}	0.034 (0.011)	0.97	5.4×10^{-01}	0.045 (0.011)	1.29	1.7×10^{-01}
CD	0.18	0.20	0.18	0.037	0.055 (0.006)	1.39	5.7×10^{-03}	0.059 (0.006)	1.48	1.3×10^{-03}	0.054 (0.006)	1.37	6.6×10^{-03}
HT	0.60	0.82	0.71	0.012	0.025 (0.010)	1.67	1.7×10^{-01}	0.022 (0.012)	1.38	3.0×10^{-01}	0.024 (0.011)	1.58	2.0×10^{-01}
RA	0.11	0.17	0.12	0.008	0.012 (0.004)	1.28	2.6×10^{-01}	0.010 (0.004)	1.09	4.2×10^{-01}	0.011 (0.004)	1.27	2.7×10^{-01}
T1D	0.13	0.16	0.13	0.016	0.026 (0.005)	1.52	4.6×10^{-02}	0.025 (0.006)	1.45	7.8×10^{-02}	0.025 (0.005)	1.42	8.0×10^{-02}
T2D	0.36	0.55	0.42	0.039	0.056 (0.012)	1.31	1.4×10^{-01}	0.053 (0.014)	1.19	2.7×10^{-01}	0.050 (0.012)	1.16	2.9×10^{-01}
UC	0.17	0.25	0.21	0.012	0.022 (0.003)	1.63	5.0×10^{-03}	0.024 (0.004)	1.68	3.8×10^{-03}	0.022 (0.003)	1.56	7.2×10^{-03}
MS	0.19	0.26	0.21	0.012	0.037 (0.003)	2.12	5.5×10^{-11}	0.041 (0.004)	2.07	6.5×10^{-09}	0.034 (0.003)	1.86	8.8×10^{-08}
Average:						1.44			1.29			1.34	
