# Supplementary material for: Quantifying Missing Heritability at Known GWAS Loci
Source: PLoS Genet. 2013 Dec 26;9(12):e1003993. doi: 10.1371/journal.pgen.1003993 (PMC3873246; doi:10.1371/journal.pgen.1003993)
Supplement: Table S19 — Heritability of known autoimmune disease loci. Local heritability inferred by LD-adjusted variance-components is reported for known loci associated with autoimmune disease (but not associated for the focal trait). computed from genome-wide and % of genome. P-value computed for versus using analytical standard error. (PDF) [file pgen.1003993.s027.pdf]

Table S19. Heritability of known autoimmune disease loci.

Phenotype	Total $h^2_{g\text{LD}}$	Loci	% genome	h^2_{gLD} Local			
				h^2_{null}	h^2_{gLD} (se)	$h^2_{\text{gLD}}/h^2_{\text{null}}$	P-Value
Autimmune Traits:							
MS	0.26	251	6.8%	0.018	0.046 (0.005)	2.59	1.9×10^{-09}
UC	0.25	129	4.3%	0.011	0.032 (0.007)	2.90	1.1×10^{-03}
CD	0.20	26	1.0%	0.002	0.024 (0.005)	11.90	8.2×10^{-06}
RA	0.17	24	0.9%	0.002	0.014 (0.006)	8.95	1.4×10^{-02}
T1D	0.16	24	0.9%	0.002	0.011 (0.005)	7.57	3.2×10^{-02}
							Combined: 5.0×10^{-15}
Other Traits:							
BD	0.27	30	1.1%	0.003	0.005 (0.006)	1.48	4.0×10^{-01}
CAD	0.31	28	1.1%	0.003	0.001 (0.009)	0.28	6.0×10^{-01}
HT	0.82	32	1.2%	0.010	0.023 (0.015)	2.31	1.9×10^{-01}
T2D	0.55	34	1.3%	0.007	0.000 (0.012)	0.00	7.1×10^{-01}
							Combined: 4.4×10^{-01}
