# Supplementary material for: Quantifying Missing Heritability at Known GWAS Loci
Source: PLoS Genet. 2013 Dec 26;9(12):e1003993. doi: 10.1371/journal.pgen.1003993 (PMC3873246; doi:10.1371/journal.pgen.1003993)
Supplement: Table S20 — Effect of LD adjustment on heritability of autoimmune disease loci. Results from three methods for estimating local variance-components are reported, (standard), (LD-residual adjusted), and (LDAK adjusted). Gain column reports corresponding , where is computed based on the genome-wide and locus size. P-value computed from z-test using analytical standard error. (PDF) [file pgen.1003993.s028.pdf]

Table S20. Effect of LD adjustment on heritability of autoimmune disease loci.

				Unadjusted			LD-residual Adjusted			LDAK Adjusted		
Phenotype	h^2_{g}	h^2_{gLD}	h^2_{gLDAK}	$h^2_{\text{g,local}}$ (se)	Gain	P-value	$h^2_{\text{gLD,local}}$ (se)	Gain	P-value	$h^2_{\text{gLDAK,local}}$ (se)	Gain	P-value
Autoimmune Traits:												
MS	0.19	0.26	0.21	0.039 (0.004)	3.04	9.9×10^{-14}	0.046 (0.005)	2.59	1.9×10^{-09}	0.040 (0.004)	2.74	1.2×10^{-11}
UC	0.17	0.25	0.21	0.029 (0.005)	3.88	5.4×10^{-06}	0.032 (0.007)	2.90	1.1×10^{-03}	0.029 (0.005)	3.17	1.2×10^{-04}
CD	0.18	0.20	0.18	0.022 (0.005)	12.38	5.3×10^{-06}	0.024 (0.005)	11.90	8.2×10^{-06}	0.022 (0.005)	12.51	6.6×10^{-06}
RA	0.11	0.17	0.12	0.011 (0.005)	10.77	2.1×10^{-02}	0.014 (0.006)	8.95	1.4×10^{-02}	0.014 (0.005)	12.88	4.7×10^{-03}
T1D	0.13	0.16	0.13	0.012 (0.004)	10.04	5.5×10^{-03}	0.011 (0.005)	7.57	3.2×10^{-02}	0.010 (0.005)	8.05	3.0×10^{-02}
Average:					8.02			6.78			7.87	
Other Traits:												
BD	0.26	0.27	0.28	0.008 (0.005)	2.47	1.7×10^{-01}	0.005 (0.006)	1.48	4.0×10^{-01}	0.005 (0.005)	1.59	3.7×10^{-01}
CAD	0.30	0.31	0.30	0.001 (0.006)	0.35	6.3×10^{-01}	0.001 (0.009)	0.28	6.0×10^{-01}	0.001 (0.009)	0.26	6.1×10^{-01}
HT	0.60	0.82	0.71	0.004 (0.011)	0.35	7.3×10^{-01}	0.023 (0.015)	2.31	1.9×10^{-01}	0.015 (0.015)	1.53	3.6×10^{-01}
T2D	0.36	0.55	0.42	0.008 (0.009)	1.11	4.7×10^{-01}	0.000 (0.012)	0.00	7.1×10^{-01}	0.002 (0.010)	0.25	6.9×10^{-01}
Average:					1.27			1.02			0.98	
