# Supplementary material for: Quantifying Missing Heritability at Known GWAS Loci
Source: PLoS Genet. 2013 Dec 26;9(12):e1003993. doi: 10.1371/journal.pgen.1003993 (PMC3873246; doi:10.1371/journal.pgen.1003993)
Supplement: Table S21 — Heritability of autoimmune disease loci adjusted for genic enrichment. Enrichment of GWAS loci at genic regions is quantified and adjusted for by recomputing as weighted average of genic and non-genic region size and corresponding total genome-wide . Total computed from two variance-components for genic and non-genic regions modeled jointly. P-value computed for versus adjusted using analytical standard error. (PDF) [file pgen.1003993.s029.pdf]

**Table S21. Heritability of autoimmune disease loci adjusted for genic enrichment.**

| | Fraction "genic"[a] | | Total $h^2_{\mathrm{gLD}}$ | | Local $h^2_{\mathrm{gLD}}$ | | | |
|---|---|---|---|---|---|---|---|---|
| Phenotype | Genome | GWAS Loci | Genome | Genic regions[b] | $h^2_{\mathrm{null}}$ | $h^2_{\mathrm{gLD}}$ (se) | $h^2_{\mathrm{gLD}}/h^2_{\mathrm{null}}$ | P-Value |
| Autoimmune Traits: | | | | | | | | |
| MS | 54% | 63% | 0.261 | 0.171 | 0.018 | 0.046 (0.005) | 2.49 | $4.6 \times 10^{-09}$ |
| UC | 49% | 58% | 0.252 | 0.190 | 0.012 | 0.032 (0.007) | 2.67 | $1.7 \times 10^{-03}$ |
| CD | 50% | 63% | 0.197 | 0.121 | 0.002 | 0.024 (0.005) | 11.22 | $9.1 \times 10^{-06}$ |
| RA | 50% | 66% | 0.166 | 0.084 | 0.002 | 0.014 (0.006) | 8.91 | $1.4 \times 10^{-02}$ |
| T1D | 50% | 56% | 0.163 | 0.148 | 0.002 | 0.011 (0.005) | 6.87 | $3.4 \times 10^{-02}$ |
| Other Traits: | | | | | | | | |
| BD | 50% | 60% | 0.269 | 0.157 | 0.003 | 0.005 (0.006) | 1.43 | $4.1 \times 10^{-01}$ |
| CAD | 50% | 59% | 0.308 | 0.202 | 0.004 | 0.001 (0.009) | 0.26 | $6.1 \times 10^{-01}$ |
| HT | 50% | 63% | 0.821 | 0.421 | 0.010 | 0.023 (0.015) | 2.30 | $1.9 \times 10^{-01}$ |
| T2D | 50% | 61% | 0.551 | 0.360 | 0.007 | 0.000 (0.012) | 0.00 | $7.3 \times 10^{-01}$ |

[a]Within 10kbp of known exon.

[b]Computed from joint model of one genic and one non-genic component.
