# Supplementary material for: Quantifying Missing Heritability at Known GWAS Loci
Source: PLoS Genet. 2013 Dec 26;9(12):e1003993. doi: 10.1371/journal.pgen.1003993 (PMC3873246; doi:10.1371/journal.pgen.1003993)
Supplement: Table S22 — Combined local heritability for autoimmune traits. Estimates of local heritability from known GWAS loci for the respective trait and any known loci for other autoimmune traits are presented separately and together. reports the heritability at known GWAS loci for the specified trait. for non-trait autoimmune loci is zero by definition. is computed from and genome-wide . P-values are computed by z-test using the analytical standard error on . (PDF) [file pgen.1003993.s030.pdf]

Table S22. Combined local heritability for autoimmune traits.

	Total $h^2_{g\mathrm{LD}}$	Known loci for trait				Non-trait autoimmune loci			Combined	
		h^2_{GWAS}	h^2_{null}	$h^2_{g\mathrm{LD}}$ (se)	P-value	h^2_{null}	$h^2_{g\mathrm{LD}}$ (se)	P-value	$h^2_{\mathrm{gLD}}/h^2_{\mathrm{null}}$ (P-value)	$h^2_{\mathrm{gLD}}/h^2_{\mathrm{GWAS}}$ (P-value)
CD	0.20	0.037	0.040	0.059 (0.006)	1.3×10^{-03}	0.002	0.024 (0.005)	8.2×10^{-06}	1.98 (1.2×10^{-07})	2.20 (6.1×10^{-09})
RA	0.17	0.008	0.009	0.010 (0.004)	4.2×10^{-01}	0.002	0.014 (0.006)	1.4×10^{-02}	2.19 (4.6×10^{-02})	2.80 (2.2×10^{-02})
T1D	0.16	0.016	0.018	0.025 (0.006)	7.8×10^{-02}	0.002	0.011 (0.005)	3.2×10^{-02}	1.94 (1.1×10^{-02})	2.24 (4.2×10^{-03})
UC	0.25	0.012	0.014	0.024 (0.004)	3.8×10^{-03}	0.011	0.032 (0.007)	1.1×10^{-03}	2.20 (2.4×10^{-05})	4.50 (1.1×10^{-08})
MS	0.26	0.012	0.020	0.041 (0.004)	6.5×10^{-09}	0.018	0.046 (0.005)	1.9×10^{-09}	2.31 (1.1×10^{-16})	7.15 ($< 1.0 \times 10^{-16}$)
