# Supplementary material for: Quantifying Missing Heritability at Known GWAS Loci
Source: PLoS Genet. 2013 Dec 26;9(12):e1003993. doi: 10.1371/journal.pgen.1003993 (PMC3873246; doi:10.1371/journal.pgen.1003993)
Supplement: Table S23 — Fraction of local heritability explained in RACI simulated phenotypes. Analysis of simulated disease architecture with 180 causal 1 Mbp loci yielding a true . In each locus, 1–10 causal variants were sampled from either low-frequency () of common (MAF) ImmunoChip SNPs and then hidden. For each of four methods tested, the fraction of local heritability identified by the method is reported over 30 simulations (with standard error in parenthesis). was restricted to SNPs present in WTCCC1 only (consistent with our real analysis). The gain of over is reported in the bottom row of each panel. (PDF) [file pgen.1003993.s031.pdf]

Table S23. Fraction of local heritability explained in RACI simulated phenotypes.

A:	# Low-frequency untyped causals: 1	2	3	5	10
h^2_{GWAS}	11%	11%	10%	8%	6%
$h^2_{\text{GWAS,joint}}$	39%	35%	29%	18%	28%
h^2_g local (se)	68% (1.5%)	65% (2.7%)	62% (2.5%)	56% (3.2%)	44% (2.0%)
$h^2_{g\text{LD}}$ local (se)	53% (1.2%)	57% (1.1%)	54% (1.3%)	52% (1.5%)	67% (0.9%)
$h^2_{g\text{LD}}$ local increase	4.61	4.96	5.23	5.96	10.80

B:	# Common untyped causals: 1	2	3	5	10
h^2_{GWAS}	45%	38%	33%	28%	19%
$h^2_{\text{GWAS,joint}}$	66%	56%	51%	47%	49%
h^2_g local (se)	101% (1.1%)	107% (0.9%)	72% (1.0%)	103% (0.9%)	103% (0.8%)
$h^2_{g\text{LD}}$ local (se)	67% (1.2%)	70% (1.4%)	69% (1.3%)	68% (1.0%)	69% (1.0%)
$h^2_{g\text{LD}}$ local increase	1.50	1.83	2.05	2.39	3.62

Mean and standard error (se) reported over 30 independent trials.
