# Supplementary material for: Quantifying Missing Heritability at Known GWAS Loci
Source: PLoS Genet. 2013 Dec 26;9(12):e1003993. doi: 10.1371/journal.pgen.1003993 (PMC3873246; doi:10.1371/journal.pgen.1003993)
Supplement: Table S24 — Computation of increased heritability in ImmunoChip data. Four alternative estimates of are considered for the ImmunoChip data. Top two panels show enrichment assuming total equals 0.17 as in WTCCC1 or 0.40 as in previously published estimates of . Bottom two panels show the same two assumptions with “flanking” regions estimated from tagging in 1,000 Genomes (see Results). P-value computed for versus corresponding using analytical standard error. (PDF) [file pgen.1003993.s032.pdf]

Table S24. Computation of increased heritability in ImmunoChip data.

Loci	% genome	h^2_{GWAS}	h^2_{null}	$h^2_{g\mathrm{LD}}$ local (se)	$h^2_{g\mathrm{LD}}/h^2_{\mathrm{null}}$	P-value
h^2_g total = 0.17						
RA GWAS	0.3%	0.0063	0.0067	0.014 (0.002)	2.16	9.9×10^{-06}
Autoimmune GWAS	0.6%	0.0000	0.0010	0.018 (0.002)	18.88	1.1×10^{-16}
h^2_g total = 0.40						
RA GWAS	0.3%	0.0063	0.0073	0.014 (0.002)	1.97	4.5×10^{-05}
Autoimmune GWAS	0.6%	0.0000	0.0023	0.018 (0.002)	7.84	1.3×10^{-14}
h^2_g total = 0.17 and flanking						
RA GWAS	0.4%	0.0063	0.0069	0.014 (0.002)	2.09	1.7×10^{-05}
Autoimmune GWAS	0.8%	0.0000	0.0014	0.018 (0.002)	13.37	3.3×10^{-16}
h^2_g total = 0.40 and flanking						
RA GWAS	0.4%	0.0063	0.0079	0.014 (0.002)	1.84	1.5×10^{-04}
Autoimmune GWAS	0.8%	0.0000	0.0033	0.018 (0.002)	5.55	4.2×10^{-13}
