# Supplementary material for: Quantifying Missing Heritability at Known GWAS Loci
Source: PLoS Genet. 2013 Dec 26;9(12):e1003993. doi: 10.1371/journal.pgen.1003993 (PMC3873246; doi:10.1371/journal.pgen.1003993)
Supplement: Table S25 — Heritability of previously implicated RA loci in ImmunoChip. Components of local heritability were estimated at two groups of loci suspected in previously published papers. P-value computed for versus corresponding using analytical standard error. (PDF) [file pgen.1003993.s033.pdf]

Table S25. Heritability of previously implicated RA loci in ImmunoChip.

Annotation	Samples	# Loci	% genome	h^2_{GWAS}	h^2_{GWAS} joint	$h^2_{\text{gLD,local}}$ (se)	$h^2_{\text{gLD}}/h^2_{\text{null}}$	P-Value
Eyre conditional	23,092	6	0.2%	0.006	0.011	0.017 (0.002)	2.81	1.6×10^{-07}
Diogo sequenced	23,092	9	0.3%	0.004	0.008	0.013 (0.002)	3.11	1.2×10^{-07}
