# Supplementary material for: Quantifying Missing Heritability at Known GWAS Loci
Source: PLoS Genet. 2013 Dec 26;9(12):e1003993. doi: 10.1371/journal.pgen.1003993 (PMC3873246; doi:10.1371/journal.pgen.1003993)
Supplement: Table S26 — SNPs analyzed in local variance-components. The number and allele frequency spectrum of SNPs used in local heritability analysis. For known GWAS loci, all genotyped SNPs at the locus as well as any imputed GWAS associated SNPs were included (over the seven WTCCC1 where imputation was performed). For autoimmune loci only genotyped SNPs were included. Effective number of SNPs computed as the sum of SNP variances after performing the LD residual. (PDF) [file pgen.1003993.s034.pdf]

Table S26. SNPs analyzed in local variance-components.

Known GWAS loci:							
			Allele frequency spectrum				
Phenotype	Effective	Total	(0.0-0.1]	(0.1-0.2]	(0.2-0.3]	(0.3-0.4]	(0.4-0.5]
BD	743	1560	251	367	366	269	291
CAD	862	1804	289	407	370	331	383
CD	1092	2465	408	530	574	454	455
HT	385	798	127	183	186	112	180
RA	418	886	170	216	178	163	150
T1D	617	1236	222	290	267	241	207
T2D	903	1788	320	392	355	354	343
UC	957	3269	719	734	694	575	547
MS	4259	13221	2198	3287	2812	2482	2442
RA:Immunochip	1264	11074	3339	2195	1597	1940	2003

Known autimmune loci:							
			Allele frequency spectrum				
Phenotype	Effective	Total	(0.0-0.1]	(0.1-0.2]	(0.2-0.3]	(0.3-0.4]	(0.4-0.5]
BD	986	2017	328	473	450	392	374
CAD	929	1832	328	438	408	331	327
CD	912	1837	326	430	402	351	328
HT	1066	2100	340	498	491	406	365
RA	777	1624	280	403	352	286	303
T1D	747	1546	247	367	341	297	294
T2D	1078	2145	343	499	482	424	397
UC	5288	18331	4396	3977	3590	3244	3124
MS	8842	27772	4292	6878	5963	5485	5154
RA:Immunochip	5184	46801	14718	10523	7762	7085	6713
