# Supplementary material for: Quantifying Missing Heritability at Known GWAS Loci
Source: PLoS Genet. 2013 Dec 26;9(12):e1003993. doi: 10.1371/journal.pgen.1003993 (PMC3873246; doi:10.1371/journal.pgen.1003993)
Supplement: Table S27 — Local heritability enrichment adjusted for SNP density and SNP LD. Three metrics of local expectation are considered: “Physical size”: the fraction of the physical genome taken up by the loci; “SNP size”: the fraction of SNPs within the loci; “LD residual variance”: the fraction of SNP variances in the loci after transforming to LD residuals. For each metric, the local expectation is recomputed and the resulting “Gain” () is reported with its corresponding p-value. (PDF) [file pgen.1003993.s035.pdf]

Table S27. Local heritability enrichment adjusted for SNP density and SNP LD.

Known GWAS loci:									
	Physical size			SNP size			LD residual variance		
Phenotype	% genome	Gain	P-value	% genome	Gain	P-value	% genome	Gain	P-value
BD	0.9%	0.31	9.8×10^{-01}	1.1%	0.30	9.8×10^{-01}	1.2%	0.30	9.9×10^{-01}
CAD	1.1%	0.97	5.4×10^{-01}	1.3%	0.95	5.6×10^{-01}	1.4%	0.95	5.7×10^{-01}
CD	1.4%	1.48	1.3×10^{-03}	1.7%	1.46	1.7×10^{-03}	1.7%	1.46	1.8×10^{-03}
HT	0.5%	1.38	3.0×10^{-01}	0.6%	1.30	3.3×10^{-01}	0.6%	1.27	3.5×10^{-01}
RA	0.5%	1.09	4.2×10^{-01}	0.6%	1.07	4.4×10^{-01}	0.7%	1.06	4.5×10^{-01}
T1D	0.7%	1.45	7.8×10^{-02}	0.9%	1.43	8.6×10^{-02}	1.0%	1.42	9.1×10^{-02}
T2D	1.1%	1.19	2.7×10^{-01}	1.3%	1.16	2.9×10^{-01}	1.5%	1.13	3.2×10^{-01}
UC	0.8%	1.68	3.8×10^{-03}	0.7%	1.71	3.0×10^{-03}	0.8%	1.68	3.6×10^{-03}
MS	3.1%	2.07	6.5×10^{-09}	3.3%	2.00	1.7×10^{-08}	3.6%	1.94	4.6×10^{-08}
Average		1.29	3.3×10^{-05}		1.27	7.0×10^{-05}		1.24	1.4×10^{-04}
Known autimmune loci:									
	Physical size			SNP size			LD residual variance		
Phenotype	% genome	Gain	P-value	% genome	Gain	P-value	% genome	Gain	P-value
CD	1.0%	11.90	8.2×10^{-06}	1.2%	9.59	1.3×10^{-05}	1.5%	8.25	1.8×10^{-05}
RA	0.9%	8.95	1.4×10^{-02}	1.1%	7.24	1.7×10^{-02}	1.3%	6.52	1.9×10^{-02}
T1D	0.9%	7.57	3.2×10^{-02}	1.1%	6.34	3.6×10^{-02}	1.2%	5.76	3.9×10^{-02}
UC	4.3%	2.90	1.1×10^{-03}	4.1%	3.07	8.0×10^{-04}	4.6%	2.75	1.4×10^{-03}
MS	6.8%	2.59	1.9×10^{-09}	7.0%	2.52	3.5×10^{-09}	7.5%	2.36	1.6×10^{-08}
Average		6.78	5.0×10^{-15}		5.75	1.1×10^{-14}		5.13	8.2×10^{-14}
BD	1.1%	1.48	4.0×10^{-01}	1.4%	1.20	4.5×10^{-01}	1.6%	1.07	4.8×10^{-01}
CAD	1.1%	0.28	6.0×10^{-01}	1.3%	0.23	6.3×10^{-01}	1.5%	0.20	6.6×10^{-01}
HT	1.2%	2.31	1.9×10^{-01}	1.5%	1.89	2.4×10^{-01}	1.7%	1.63	2.8×10^{-01}
T2D	1.3%	0.00	7.1×10^{-01}	1.5%	0.00	7.5×10^{-01}	1.7%	0.00	7.8×10^{-01}
Average		1.02	4.4×10^{-01}		0.83	5.3×10^{-01}		0.72	6.1×10^{-01}
