# Supplementary material for: Quantifying Missing Heritability at Known GWAS Loci
Source: PLoS Genet. 2013 Dec 26;9(12):e1003993. doi: 10.1371/journal.pgen.1003993 (PMC3873246; doi:10.1371/journal.pgen.1003993)
Supplement: Table S28 — Heritability of MS data with PCA-matched samples. Cases and controls were matched pair-wise based on top 20 principal components (retaining 8,149 samples) and components of local heritability re-estimate. (PDF) [file pgen.1003993.s036.pdf]

Table S28. Heritability of MS data with PCA-matched samples.

A: GWAS Loci							
Cohort	# Samples	Total h^2_{gLD}	h^2_{GWAS}	$h^2_{\text{GWAS,joint}}$	$h^2_{g\text{LD}}$ local (se)	$h^2_{g\text{LD}}/h^2_{\text{null}}$	P-Value
MS	14526	0.26	0.012	0.014	0.041 (0.004)	2.08	5.2×10^{-09}
MSMATCH	8149	0.29	0.023	0.027	0.053 (0.006)	1.72	4.0×10^{-05}
B: Autoimmune Loci							
Cohort	# Samples	Total h^2_{gLD}	h^2_{GWAS}	$h^2_{\text{GWAS,joint}}$	$h^2_{g\text{LD}}$ local (se)	Increase	P-Value
MS	14526	0.26	0.000	0.000	0.046 (0.005)	2.59	1.9×10^{-09}
MSMATCH	8149	0.29	0.000	0.000	0.053 (0.008)	2.67	4.7×10^{-06}
